# Higher trait working memory capacity may benefit standardized test performance under race-related stereotype threat

**DOI:** 10.3758/s13421-025-01723-y

**Published:** 2025-05-19

**Authors:** LaTasha R. Holden, Kerri A. Goodwin, Andrew R. A. Conway

**Affiliations:** 1 Department of Psychology, University of Illinois Urbana-Champaign, Champaign, IL 61820 USA; 2 Beckman Institute, Urbana, IL USA; 3 Department of Psychology, Towson University, Towson, MD USA; 4 Department of Psychology, New Mexico State University, Las Cruces, NM USA

**Keywords:** Stereotype threat, Racial/Ethnic minorities, Working memory capacity, Standardized test performance, Individual differences

## Abstract

Stereotype threat (ST) occurs when individuals primed with negative stereotypes underperform relative to a control group. Activating ST increases anxiety and worries about being negatively perceived, also introducing mental distraction that negatively impacts performance. We consider racial/ethnic ST effects on standardized test performance (SDTP) on the verbal and quantitative reasoning sections of the Graduate Record Exam (GRE). Across two experiments, working memory capacity (WMC) is investigated as a mediator and/or moderator of ST for race/ethnicity (Experiment 1, final *n* = 447, 19% Black, 81% White, 59% female and for Experiment 2, *n* = 166, 41% Black, 59% White, 73% female). We find a lack of strong evidence for the classic ST effect of a Race × Condition interaction. However, we show evidence that for Black students, higher trait WMC moderates racial/ethnic ST such that higher WMC is associated with higher scores on standardized tests under conditions of race-related ST. Our findings suggest the importance of higher WMC for racial minority students in remaining mentally resilient and maintaining performance during ST. Future work should address diversity and inclusion concerns regarding research on ST effects for racial/ethnic minorities, include more work examining racial/ethnic ST based on replication issues and statistical power, as well as more examination of the importance of WMC for performance under racial/ethnic ST. Future work should also consider the roles of protective factors, such as mindfulness and self-regulation practices in the context of racial/ethnic ST as WMC and SDTP have been shown to generally improve through implementing these practices.

**Supplementary Information:**

The online version contains supplementary material available at 10.3758/s13421-025-01723-y.

## Stereotype threat

For people who have stigmatized social or group identities, being primed with a negative stereotype has been shown to cause underperformance on standardized tests relative to a control group, an effect known as stereotype threat (ST; Steele & Aronson, 1995). As one of the most researched topics in psychology, the effect of ST on performance has been shown for ethnic/racial identity (Steele & Aronson, 1995), sex/gender (Regner et al., 2010; Schmader & Johns, 2003), socioeconomic status (Flores et al., 2018; Tine & Gotlieb, 2013), and age (Levy, 1996). Outside of the U.S. context, ST research has focused a great deal on gender threat—examining its impact on women’s performance in math or STEM domains (see Flore & Wicherts, 2014; also Huguet & Regner, 2007; Regner et al., 2010). However, if a negative stereotype exists regarding cognitive performance of certain racial/ethnic groups in other countries, then in theory, ST effects could be observed in those cases, too. In the U.S. context compared with outside the U.S., the impact racial/ethnic ST effects have on performance is complicated by differences in attributions made based on having a racialized minority status. While these are interesting questions, they remain largely outside the scope of the current work, as we will focus on investigating racial/ethnic ST effects in U.S. student samples.

Although ST has been investigated widely, we focus on the effect of ST on cognitive tasks that are important for racialized minority students’ test performance and achievement in the U.S. Using data from the National Assessment of Educational Progress (NAEP), Stanford’s Center for Education Policy Analysis suggests that gaps in achievement between minority students and White students have narrowed since the 1970 s (Reardon, 2015; also, National Center for Education Statistics, 2013). However, gaps remain in White and Black students’ standardized test performance (SDTP) for reading and math in elementary through high school (National Center for Education Statistics, 2017). In the U.S. educational system, standardized tests such as the Scholastic Assessment Test (SAT) and the Graduate Record Exam (GRE)^1^ are important for demonstrating preparation for admittance to higher education institutions. Now, many undergraduate and doctoral programs have made these tests optional, but they are still required in some places. These tests are administered to millions of students in and outside of the U.S. (ETS, 2018), and achievement gaps remain between White and Black students in the U.S. Previous work suggests that worries about being negatively stereotyped based on racial/ethnic group is enough to impact Black students’ performance on standardized tests and other academic assessments (see Steele, 1997; Steele & Aronson, 1995).

Although there are continuing debates about their utility, standardized tests still have the power to open and close doors for students, and understanding the influence(s) of the testing environment for students is essential. The pressure of performing well, coupled with other anxieties, can cause some students to “choke” and underperform. Ironically, those who are among those most capable of performing well—deemed the academic “vanguard” (Steele, 1997)—tend to underperform in high-pressure situations (cf. Beilock & Carr, 2005; Beilock & DeCaro, 2007).

These findings beg the question of what factors are most important in understanding differences in performance on assessments of cognitive ability. Suggested factors include situational or contextual influences (Massey & Owens, 2014; Mullainathan & Shafir, 2013; Steele & Aronson, 1995), practice or experience (Ericsson et al., 1993; Hambrick et al., 2016), one’s intelligence (Jensen, 1969; Kovacs & Conway, 2016; Spearman, 1904; also see Holden & Tanenbaum, 2023), working memory capacity (WMC; Baddeley & Hitch, 1974; Conway et al., 2003; Regner et al., 2010; Schmader & Johns, 2003), as well as personality and attitudinal factors (Aronson et al., 2002; Blackwell et al., 2007; Dweck & Leggett, 1988; also Duckworth & Yeager, 2015; Durlak et al., 2011; Yeager & Walton, 2011). Based on Steele and Aronson (1995), having to contend with others viewing you based on a stereotype is enough to negatively shift your performance; however, the phenomenological process of ST is quite complicated and involves many integrated cognitive, affective, and physiological processes (Schmader et al., 2008). In terms of the cognitive mechanisms involved in ST, previous research suggests the importance of cognitive control or our ability to focus our attention and mental resources on completing a task goal (see Spencer et al., 2016).

When handling the cognitive demands of ST, people can be impacted in their ability to focus and control their mental resources. When a negative stereotype is made salient for a certain group, those identified with that group may have concerns about performing in way that would “prove” the stereotype. Considering this, people are motivated to avoid “confirming” the stereotype (see Steele, 1997) which could involve putting forth extra effort. Work by Jamieson and Harkins (2007) showed that participants under ST had difficulty inhibiting automatic responses on the antisaccade task but were able to quickly correct their responses. These findings suggest that although the antisaccade task was challenging under ST, participants were not overloaded cognitively in that they were still able to update their responses from incorrect to correct.

Additional work has suggested that when we are faced with challenging cognitive tasks and experiencing ST, we are contending with the stress and worries regarding ST as well as regulating ourselves to focus our attention and mental resources on completing the task at hand (see Beilock et al., 2007; Jamieson & Harkins, 2007; Regner et al., 2010; Schmader et al., 2008; Schmader & Johns, 2003; Steele, 1997). This means that ST can introduce an additional form of mental load on top of the challenges associated with solving difficult problems alone. Overall, much of previous research examining the cognitive mechanisms involved in ST suggests that cognitive control abilities, like the capacity of working memory, are of great importance. Activating ST may cause people to have difficulty with inhibiting incorrect responses, and especially so when the task is very challenging. This means the impact of differences in cognitive control and capacity of working memory on SDTP in the context of ST is important to understand. Next, we will consider the consequences of ST in terms of SDTP, then we will discuss the role of cognitive control through differences in working memory.

## Consequences of stereotype threat

A key component of ST theory is about the *consequences* of being the target of stereotyping, including effects on assessments of cognitive ability, like standardized tests. Although the emphasis of standardized tests for placement and acceptance into academic programs has been criticized and has decreased some over time (Ramirez, 2008; Serrano, 2015), generally, such tests are still utilized and often weighted heavily in admissions decisions (Lauryn, 2017; National Association for College Admission Counseling, 2016). For these reasons, additional research is needed to clarify influences during high-stakes testing for targets of stereotyping and ST. Because our focus is on the cognitive performance responsible for differences in standardized test performance, we discuss two perspectives from the literature that involve the ways ST impacts cognitive resources through WMC.

## Stereotype threat and working memory capacity

One perspective is that experiencing threats to group identity causes a reduction in the ability to focus on the task at hand. Working memory is an established construct in the literature that taps our ability to focus attention on a task, while simultaneously storing, retrieving, and updating other key information (Baddeley, 1996, 2000; Baddeley & Hitch, 1974). Working memory (WM) is thought to have a capacity component (called working memory capacity, or WMC) that varies across individuals and constrains the parameters by which people activate and utilize the cognitive resources at their disposal (Cowan, 1988; Daneman & Carpenter, 1980; Turner & Engle, 1989). When considering the role of cognitive resources during identity-threatening situations, Schmader and Johns (2003) found that ST causes a depletion in the form of lower WMC. Further, they characterized this finding as a decrease in the ability to regulate one’s behavior in a goal-oriented way (also see Schmader et al., 2008). Others have suggested a state of mental depletion spills over and disrupts later task performance (Inzlicht & Schmeichel, 2012). For example, in several studies, under ST for either race or gender, WMC was shown to decrease compared with a control condition, and WMC mediated ST for gender on SDTP in math (Schmader & Johns, 2003). Thus, ST for gender was at least partially explained by differences in WMC.

Another perspective is that threats to group identity are moderated by the cognitive resources available at baseline. This view does not posit that threat is negated altogether; instead, it suggests when differences in trait cognitive resources are higher, they help combat identity-threatening situations. When considering baseline WMC during gender ST, for those with higher trait WMC, there was no difference in women’s and men’s fluid reasoning performance—under threat or in a control condition (Regner et al., 2010). However, Regner et al. (2010) found that when baseline WMC was lower, female students performed worse under threat compared with females under no-threat and worse than male students under threat. These results underscore the importance of higher WMC when navigating identity-threatening situations, specifically during contexts when the implications for demonstrating one’s cognitive ability is of great consequence, like with standardized tests.

## The current work

The extent to which ST effects observed in the lab generalize to actual testing situations is difficult to assess (see Steele, 1997). Although a large body of work replicates ST effects, there have been issues with researcher degrees of freedom (see Simmons et al., 2011; Wicherts et al., 2016, on topics of researcher degrees of freedom and replication issues, generally) and replication in the ST literature (Inzlicht, 2016; Schimmack, 2017; also see Flore & Wicherts, 2014; Ganley et al., 2013; Sackett et al., 2004; Shewach et al., 2019; Wicherts, 2005). Nevertheless, the fact that threat effects have been shown to negatively impact some of the most capable students is alarming. Such issues necessitate further research to clarify boundary conditions or moderators of ST as well as the mechanisms underlying the effect (for review, see Beilock et al., 2007; Spencer et al., 2016; Wheeler & Petty, 2001). To date, many investigations of ST for race have focused on group-level effects (e.g., Black vs. White students), neglecting important individual differences that may mediate and/or moderate the effect. Also, to our knowledge, most studies designed to investigate the role of WMC focused on ST effects for gender, not race (Regner et al., 2010; Schmader & Johns, 2003). Work on the role of WMC for ST for race/ethnicity has been limited to White and Latino students’ performance and has explored the role of WMC as a mediator, not a moderator (cf. Schmader & Johns, 2003). We aim to address the extent to which individual differences in WMC mediate and/or moderate ST effects for race focused on Black students’ performance.

Based on previous approaches we hypothesized that under ST for race, Black students’ performance would be lower than White students’ on state measures of WMC and on all measures of SDTP (Hypothesis 1). Second, when WMC is assessed as a trait measure, it would moderate ST such that higher trait WMC Black students would be better equipped to combat ST and perform better than Black students with lower trait WMC (Hypothesis 2). Third, we hypothesized that for WMC assessed as a state measure, ST should depress Black students’ performance as a reduction in WMC that would at least partially explain the ST effect on SDTP (Hypothesis 3). These questions were tested across two experiments conducted at two universities.

## Experiment 1

Experiment 1 considers a moderated mediation model of the role of WMC in ST effects on standardized test performance. Specifically, we tested the roles of inter- and intraindividual differences in trait and state WMC and how and when they protect students from ST during testing situations.

### Method

#### Participants

We recruited 469 undergraduates from a private university. Eleven were removed with list-wise deletion due to missing variables (e.g., in cases such as computer errors or missing portions of the standardized test worksheets). The remaining total was 458 (270 women, 188 men) native English-speaking students, ages 18 and older. Participants self-identified as White (366 students, 202 women) or Black (87 students, 66 women). For participating, students received course credit or $16 cash.

#### Design

We used a 2 (condition: threat vs. control) × 2 (WMC span task order: verbal capacity & intelligence vs. math capacity & intelligence) × 2 (race: White vs. Black) factorial design, where participants were randomly assigned to either ST or control conditions and to either WMC span task order.

##### Stereotype threat manipulation

The threat manipulation procedure was based on Schmader and Johns (2003), with slight alterations: We included a measure of WMC before and after the threat manipulation.^2^ To make the present study amenable to threat effects for both operation (OSPAN; Turner & Engle, 1989; Unsworth et al., 2005) and reading (RSPAN; Daneman & Carpenter, 1980) spans, we induced the race prime before having participants complete the working memory tasks. For OSPAN and RSPAN, participants were informed that the task was indicative of quantitative or verbal capacity, respectively, and was highly related to measures of intelligence. Moreover, they were informed that performance is attributable to group membership. Following this, they completed an ethnicity survey, which was one item to indicate one’s race (see Supplemental Methodology). The control condition did not receive any directly threatening instructions and instead were told they would only complete a working memory task.

##### Order manipulation

**Fig. 1:**
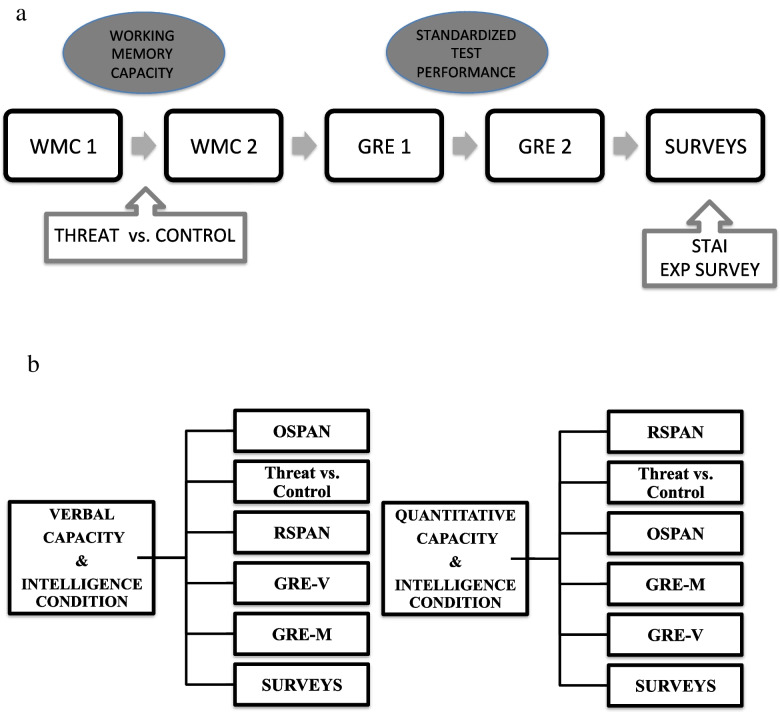
Experimental procedure. Panel **A** demonstrates the order of the tasks including the variables characterized as WMC and standardized test performance. Panel **B** demonstrates the procedures for the verbal capacity & intelligence and quantitative capacity & intelligence conditions

 Following Schmader and Johns (2003), we used an assessment of WMC to replicate the effect of threat on WMC and to test the hypothesis that state WMC mediates the effect of threat on standardized tests. To detect these effects, WMC must be measured post threat manipulation. However, we wanted to test the hypothesis that trait WMC moderates the effect of threat on standardized testing. To test this, WMC must be measured before the threat manipulation. Thus, half the participants performed RSPAN (reading) before the threat manipulation and OSPAN (math) after the threat and for the other half, vice versa. The quantitative capacity threat and intelligence group is analogous to Schmader and Johns, where OSPAN was administered post threat manipulation (see Fig. 1B).

#### Measures

##### Working memory capacity

To assess trait and state WMC, participants completed the automated OSPAN and RSPAN tasks (see Unsworth et al., 2005). OSPAN requires completing a series of arithmetic problems while remembering a list of letters. Participants solve a math problem for accuracy and then remember a letter for later recall. At the end of a list of trials, participants recall the letters in serial order (the total score was calculated using the partial unit method; see Conway et al., 2005). The RPSAN replaces math problems with making veridical judgments for sentences while remembering lists of letters. Participants received three to seven letters per trial and three sets of each trial length, totaling 15 trials—yielding a maximum score of 75.

##### Standardized test performance 

Participants completed Math and Verbal sections of the Graduate Record Exam (GRE). The GRE mathematics subsection consisted of 25 multiple-choice or short-answer questions, each requiring mathematical reasoning and quantitative comparison skills. The GRE verbal subsection was the same length but contained verbal questions requiring the abilities to analyze, evaluate, and synthesize written material, in addition to recognizing relationships among words and concepts. Both sections were timed at 20 min and taken from free online practice materials provided by the Educational Testing Service (2018). The final score was the proportion of questions correct out of 25.

##### STAI 

The Speilberger State-Trait Anxiety Inventory (STAI) short form (Marteau & Bekker, 1992) assessed state anxiety. The short form consisted of six questions, where participants indicated their present feelings using a 4-point scale ranging from 1 (*not at all*) to 4 (*very much*) to items such as “I feel calm.” Low levels of anxiety (e.g., “I am relaxed”) were reverse scored. A total score was obtained, with higher scores indicating greater anxiety.

##### Postexperiment survey 

Everyone completed a survey to mimic the ethnicity question for those in the control group and to gain a general assessment of experiences in the study (e.g., “Do you know anyone else in this study?” or “Did you feel the study was too long?”).

#### Procedure

Participants were tested in groups of up to six people. Participants completed the trait WMC span task, then received either threat or control instructions followed by a second, state WMC task. Participants who performed RSPAN before the threat manipulation completed OSPAN after the threat manipulation and vice versa. In the threat condition, participants received instructions similar to those of Schmader and Johns (2003) and completed the “ethnicity survey,” which served to prime race and induce ST. In the control condition, participants received similar instructions, which were modified to exclude the race prime.

Following the second WMC span task, all participants completed two sections of the GRE: one verbal and one quantitative section (ordered by task condition). After the GRE sections, participants completed STAI (Marteau & Bekker, 1992). Then, participants completed the postexperiment survey (this included a race prime question for those in the control condition). Lastly, participants were debriefed and thanked for their participation.

### Results

#### Statistical power

We aimed for adequate statistical power of at least 80%, based on effect sizes, as demonstrated in previous ST research. This was not straightforward, as researchers have computed effect-size estimates in different ways—some based on adjusted or unadjusted means (see Sackett et al., 2004; Shewach et al., 2019; Spencer et al., 2016; Wicherts, 2005). Additional work also suggests that the effects sizes in the literature may be inflated due to publication bias (Flore & Wicherts, 2014). We focused on recent reports on ethnic/racial ST for cognitive ability (Spencer et al., 2016). We then used the largest (*d* = 0.52) and smallest (*d* = 0.46) average effect sizes reported to estimate the sample size needed for a minimum of 80% statistical power. Using the powerInteract function in the powerMediation package in R (Qiu & Qiu, 2018), we estimated the sample size required for adequate statistical power based on a Race (White vs. Black) × Condition (threat vs. control) interaction effect,^3^ such that Factor A = 2 levels and Factor B = 2 levels for a larger effect size of Cohen’s *d* = 0.52, required a total *n* = 160 (at least 40 cases per cell); for alpha = 0.05 for a two-tailed test we would have statistical power of Beta = 0.824. For an average difference of Cohen’s *d* = 0.46, we would have statistical power of Beta = 0.806, requiring a total *n* = 120 (at least 30 cases per cell), for alpha = 0.05 for a two-tailed test. Based on the suggestion that the average effect sizes are inflated, we used the smaller average effect size reported in the literature to motivate the decision of recruiting at least 30 cases per cell in both Experiments 1 and 2. We strived to obtain statistical power based on the aforementioned calculation. Our efforts were limited by both resource and time constraints, and thus the final sample sizes obtained for Experiments 1 and 2 are reported in each Results section below.^4^ To address power concerns, we will also present results of combining samples from Experiments 1 and 2 in addition to running Bayesian regressions.

#### Data preparation and analytic approach

Upon examination of the distributions of data, additional participants were removed on the basis of being identified as univariate outliers falling outside the 3 standard deviation range of the mean for all measures of WMC and GRE. Thus, the following analyses were conducted with a final sample of 447 participants who were White (360 total, 198 women) or Black (87 total, 66 women).

Below, we present two sets of results, one for each task order condition. Because we manipulated ST for either verbal or quantitative capacity in the threat condition, we separate the threat effects based on these task orders (see Fig. 1B).

Where appropriate, the homogeneity of variance assumptions were tested with Levene’s test and when significant, Bonferroni corrections were reported. Cases where Levene’s tests were nonsignificant, normal *t* tests and analyses of variance (ANOVAs) were reported. In each regression analysis, levels of condition were dummy coded with control = 0, threat = 1. Because the threat effect was expected to manifest as a performance decrement for Black students, only their data were analyzed for WMC mediation and moderation on standardized tests.

#### Summary statistics and correlations

Descriptive statistics and correlations are reported in Table 1. The measures of WMC (i.e., OSPAN and RSPAN) were strongly positively correlated with each other as well as moderately positively correlated with measures of SDTP (i.e., math and verbal GREs). Also revealed were that higher scores on anxiety negatively correlated with most of the performance measures.

**Table 1:** Experiment 1 summary statistics

	1	2	3	4	5	6	7
1. ospan	--						
2. rspan	.590***	--					
3. gremProp	.260***	.270***	--				
4. grevProp	.220***	.270***	.465***	--			
5. gremAttProp	−0.007	.080	.601***	.186***	--		
6. grevAttProp	.021	.113*	.246***	.405***	.425***	--	
7. STAI	−0.056	-.103*	-.209***	-.092*	-.175***	−0.023	--
M	63.090	58.650	0.56	0.60	0.82	0.94	12.66
SD	10.32	11.75	0.19	0.20	0.15	0.10	3.61

The top half of the table reports Pearson’s *r *correlations and the bottom half reports *M* and *SD* for Experiment 1 measures

*ospan* Operation Span, *rspan* Reading Span, *gremProp* Proportion Correct on Math GRE, *grevProp* Proportion Correct on Verbal GRE, *gremAttProp* Proportion Attempted on Math GRE, *grevAttProp* Proportion Attempted on Verbal GRE, *STAI* Speilberger State-Trait Anxiety Inventory

***p <.001, **p <.01, *p <.05.

#### Quantitative capacity and intelligence condition

##### Baseline comparison 

An independent-samples *t* test indicated a significant difference in baseline RSPAN, *t*(197) = − 2.03, *p* = 0.044, Cohen’s *d* = 0.34, revealing that Black students (*M* = 56.27, *SD* = 12.37) scored significantly lower on RSPAN at baseline relative to White students^5^ (*M* = 60.10, *SD* = 10.78).

##### Threat effects^6^

To test the effect of ST on state WMC and SDTP, we conducted a series of 2 (race) × 2 (condition) ANOVAs. The result of interest is the interaction effect of the race and condition variables (which would indicate ST) on both students’ WMC and standardized test performance. We found none of the Race × Condition interaction effects to be significant in the quantitative capacity and intelligence condition (see Table 2). The main effects for WMC and SDTP are next reported. The effect of ST on OSPAN revealed nonsignificant effects of condition, *F*(1, 195) = 0.66, *p* = 0.42, η_p_^2^ = 0.0034,* b* = *− *1.11, *t*(195) = − 0.63, CI_95%_ [− 4.58, 2.36], *p* > 0.05^7^ and race, *F*(1, 195) = 2.3, *p* = 0.13, η_p_^2^ = 0.012, *b* = *− *2.61, *t*(195) = − 1.04, CI_95%_ [− 7.58, 2.36], *p* > 0.05.

**Table 2:** Experiment 1 Stereotype Threat Effects

Experiment 1 Analysis of Variance Summary Table											
Dependent Measure	Quantitative Capacity and Intelligence Condition					Race by Condition Interaction Effect					
	White Students			Black Students							
	Threat	Control		Threat	Control	*F*	*p*	partial *η*2	*b*	*t*	*CI* 95%
	M (SD) (*n* = 80)	M (SD) (*n* = 74)		M (SD) (*n* = 20)	M (SD) (*n* = 25)						
OSPAN	63.74 (11.41)	64.85 (9.87)		60.50 (13.81)	62.24 (9.44)	0.028	=.87	0.00015	−0.63	−0.17	[−7.95, 6.70]
GRE-M	.58 (.19)	.57 (.19)		.50 (.19)	.41 (.15)	1.67	=.20	0.0085	0.081	1.29	[-.042,.20]
GRE-V	.62 (.20)	.62 (.19)		.55 (.16)	.48 (.17)	1.14	=.27	0.0058	0.069	1.068	[-.058,.20]
Dependent Measure	Verbal Capacity and Intelligence Condition					Race by Condition Interaction Effect					
	White Students			Black Students							
	Threat		Control	Threat	Control	*F*	*p*	partial *η*2	*b*	*t*	*CI* 95%
	M (SD) (*n* = 87)		M (SD) (*n* = 119)	M (SD) (*n* = 21)	M (SD) (*n* = 21)						
RSPAN	59.78 (11.94)		58.92 (11.88)	52.57 (12.63)	53.05 (11.92)	0.11	=.74	.00045	−1.34	−0.33	[−9.34, 6.66]
GRE-V	.62 (.22)		.64 (.21)	.53 (.16)	.47 (.17)	1.2	=.27	0.0049	0.077	1.096	[-.061,.22]
GRE-M	.61 (.18)		.58 (.18)	.47 (.19)	.46 (.21)	0.053	=.81	0.00022	−0.015	−0.23	[-.14,.11]

*OSPAN* Operation Span, *RSPAN* Reading Span, *GRE-M* Proportion Correct on Math GRE, *GRE-V* Proportion Correct on Verbal GRE.

The effect of threat on the GRE-M indicated a main effect of race, *F*(1, 195) = 14.24, *p* = 0.00021, η_p_^2^ = 0.066, *b* = *− *0.15, *t*(195) = − 3.59, CI_95%_ [− 0.24, − 0.069], *p* < 0.001, where White students (*M* = 0.57, *SD* = 0.19) significantly outperformed Black students (*M* = 0.45, *SD* = 0.17). The effect of condition was nonsignificant, *F*(1, 195) = 1.09, *p* = 0.29, η_p_^2^ = 0.0055, *b* = 0.0090, *t*(195) = 0.31, CI_95%_ [− 0.049, 0.067], *p* > 0.05.

Next, the effect of threat on GRE-V, indicated a main effect of race, *F*(1, 195) = 11.57, *p* = 0.00081, η_p_^2^ = 0.055, *b* = *− *0.14, *t*(195) = − 3.21, CI_95%_ [− 0.23, − 0.054], *p* < 0.01, with White students (*M* = 0.62, *SD* = 0.20) significantly outperforming Black students (*M* = 0.51, *SD* = 0.17). The effect of condition was nonsignificant, *F*(1, 195) = 0.12, *p* = 0.73, η_p_^2^ = 0.00060,* b* = *− *0.0063, *t*(195) = − 0.21, CI_95%_ [− 0.067, 0.054], *p* > 0.05.

##### Mediation and moderation 

Due to the absence of ST in the form of a significant interaction between race and condition, there was no effect to mediate. However, we ran the analyses as planned and refer the reader to the Supplemental Information.

To test whether trait WMC (measured with RSPAN) moderates ST on standardized testing in the quantitative capacity and intelligence threat condition, hierarchical regressions were conducted with the outcome measures of math and verbal GRE scores with only Black students. In the first step, RSPAN (mean centered) and condition were entered. In the second step, the product of RSPAN (mean centered) and condition (dummy coded with control = 0, threat = 1) was added to test for moderation.

For math GRE, in Step 1, the effect of RSPAN was marginally significant at the 0.08 level, *b* = 0.0036, *t*(42) = 1.77, *p* = 0.08, indicating that higher scores on RSPAN predicted higher scores on the math GRE at the 0.08 level. The effect of condition, *b* = 0.073, *t*(42) = 1.48, *p* = 0.15, was not significant, but the model was significant, *F*(2, 42) = 3.27, Multiple *R*^2^ = 0.135, *p* = 0.048.

**Fig. 2:**
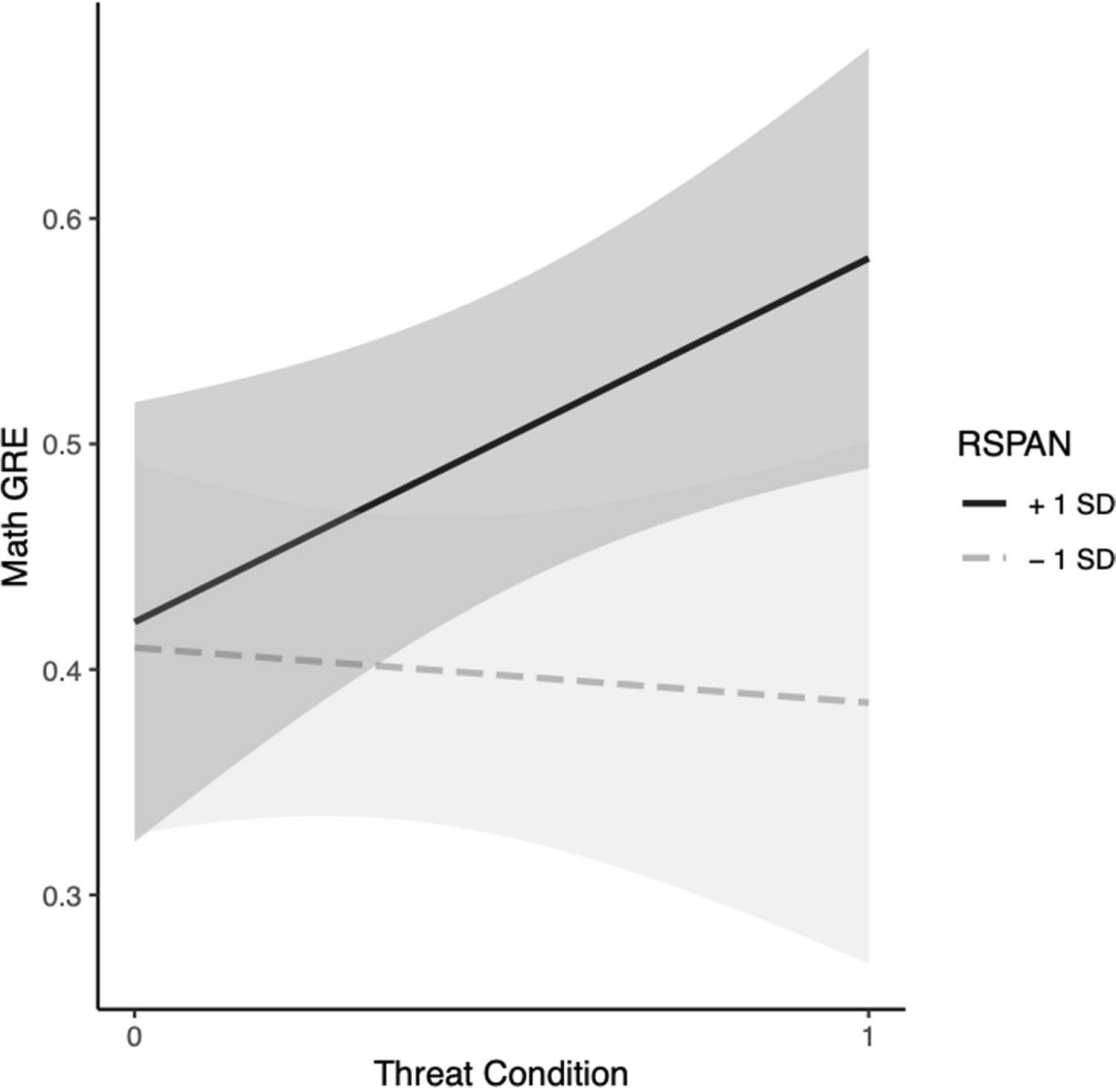
Black students’ WMC (via RSPAN) moderation model for threat on GRE-M. *Note.* Grey bands represent 95% confidence intervals

In Step 2, the effect of RSPAN, *b* = 0.00046, *t*(41) = 0.18, *p* = 0.86, and the effect of condition, *b* = *− *0.35, *t*(41) = − 1.54, *p* = 0.13, were not significant. The interaction, *b* = 0.0075, *t*(41) = 1.90, *p* = 0.065, approached significance. The change in model variance between Steps 1 and 2 also approached significance at the 0.065 level, *F*(1, 41) = 3.59,* p* = 0.065. Simple slopes (SS) tests revealed that when participants are under threat higher-WMC participants had higher predicted scores on the math GRE compared with those with lower WMC,* b* = 0.0080, *p* = 0.012, whereas, there was no significant change in GRE based on higher or lower WMC in the control condition, *b* = 0.0005, *p* = 0.86 (see Fig. 2). The model was also significant, *F*(3, 41) = 3.51, Multiple *R*^2^ = 0.20, *p* = 0.024.

**Fig. 3:**
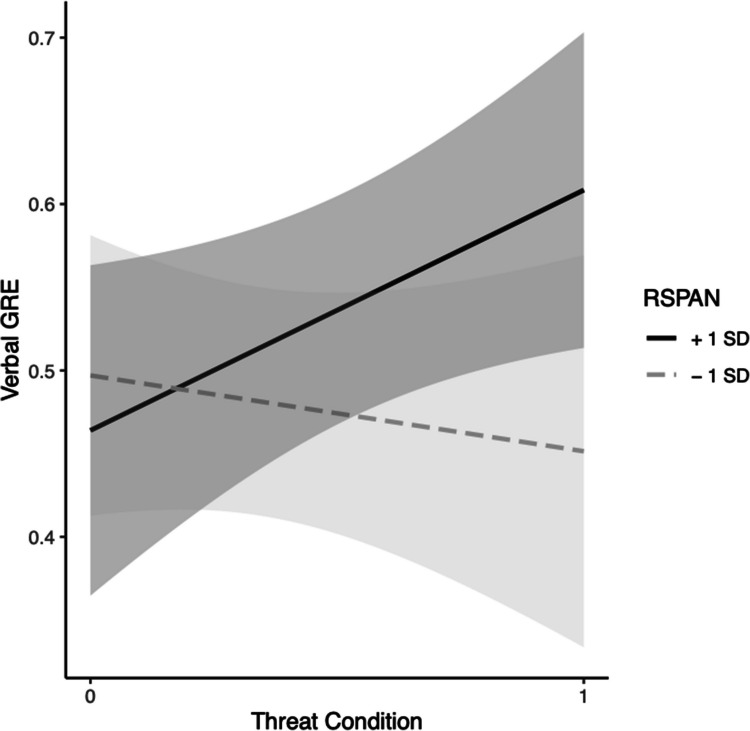
Black students’ WMC (via RSPAN) moderation model for threat on GRE-V. *Note*. Grey bands represent 95% confidence intervals

For verbal GRE, in Step 1, neither the effect of RSPAN, *b* = 0.0018, *t*(42) = 0.90, *p* = 0.38, the effect of condition, *b* = 0.054, *t*(42) = 1.081, *p* = 0.29, nor the model were significant, *F*(2, 42) = 1.21, Multiple *R*^2^ = 0.054, *p* = 0.31. In Step 2, the effects of RSPAN, *b* = *− *0.0013, *t*(41) = − 0.52, *p* = 0.61, and condition, *b* = *− *0.38, *t*(41) = − 1.63, *p* = 0.11, were nonsignificant. However, their interaction, *b* = 0.0077, *t*(41) = 1.91, *p* = 0.063, approached significance. The change in variance accounted for between models also approached significance at the 0.06 level, *F*(1, 41) = 3.63,* p* = 0.064. Simple slopes tests indicated that under threat, higher WMC participants had higher predicted scores on the verbal GRE compared with those with lower WMC,* b* = 0.0063, *p* = 0.046. There was no significant change in predicted GRE scores in the control condition, *b* = − 0.0013, *p* = 0.61. (see Fig. 3) and the model was not significant, *F*(3, 41) = 2.06, Multiple *R*^2^ = 0.13, *p* = 0.12.

#### Verbal capacity and intelligence condition

##### Baseline comparison

An independent-samples *t* test indicated a significant difference in OSPAN based on race, *t*(246) = − 2.49, CI_95%_ [− 7.35, − 0.85], *p* = 0.014, Cohen’s *d* = 0.42, with White students significantly outperforming (*M* = 63.34, *SD* = 9.58) Black students (*M* = 59.24, *SD* = 10.50) by almost a half standard deviation difference.^8^

##### Threat effects^9^

 We tested the effect of ST on state WMC and SDTP and found none of the interaction effects to be significant in the verbal capacity and intelligence condition (see Table 2). On the RSPAN we found a main effect for race, *F*(1, 244) = 10.20,* p* = 0.0016, η_p_^2^ = 0.041, *b* = − 5.87, *t*(244) = − 2.07, CI_95%_ [− 11.44, − 0.28], *p* < 0.05,^10^ such that White students performed higher (*M* = 59.28, *SD* = 11.89) than Black students (*M* = 52.81, *SD* = 12.13). The effect of condition, *F*(1, 244) = 0.17, *p* = 0.68, η_p_^2^ = 0.00070, *b* = 0.87, *t*(244) = 0.51, CI_95%_ [− 2.46, 4.19], *p* > 0.05, was nonsignificant.

For the GRE-V scores, Levene’s test revealed a significant homogeneity of variance violation, *F*(3, 244) = 3.4, *p* = 0.018. More conservative criteria for significance were used in the ANOVA model below—based on making six pairwise comparisons, a Bonferroni correction was used to compute the new significance criteria of 0.0083. Results indicated only a significant main effect of race, *F*(1, 244) = 13.87, *p* = 0.00024, η_p_^2^ = 0.053, *b* = *− *0.17, *t*(244) = − 3.42, CI_95%_ [− 0.26, − 0.071], *p* < 0.0083, such that White students (*M* = 0.63, *SD* = 0.21) significantly outperformed Black students (*M* = 0.50, *SD* = 0.17) on the verbal GRE. The effect of condition, *F*(1, 244) = 0.10, *p* = 0.75, η_p_^2^ = 0.00041, *b* = *− *0.022, *t*(244) = − 0.75, CI_95%_ [− 0.079, 0.036], *p* > 0.0083, was nonsignificant.

For GRE-M, the main effect of race was significant, *F*(1, 243) = 16.99, *p* = 0.000052, η_p_^2^ = 0.067, *b* = *− *0.13, *t*(243) = − 2.84, CI_95%_ [− 0.21, − 0.038], *p* < 0.01 such that White students (*M* = 0.60, *SD* = 0.18) significantly outperformed Black students (*M* = 0.47, *SD* = 0.20). The effect of condition was nonsignificant, *F*(1, 243) = 1.12, *p* = 0.29, η_p_^2^ = 0.0046, *b* = 0.027, *t*(243) = 1.057, CI_95%_ [− 0.024, 0.080], *p* > 0.05.

##### Mediation and moderation

Due to the absence of a ST in the form of a significant interaction between race and condition, there was no effect to mediate. However, we ran the analyses as planned and report the results in the Supplemental Information.

To test whether trait WMC (on OSPAN) moderates the effect of ST on standardized test performance, hierarchical regressions were conducted for Black students with the outcomes of verbal and math GREs.

**Fig. 4:**
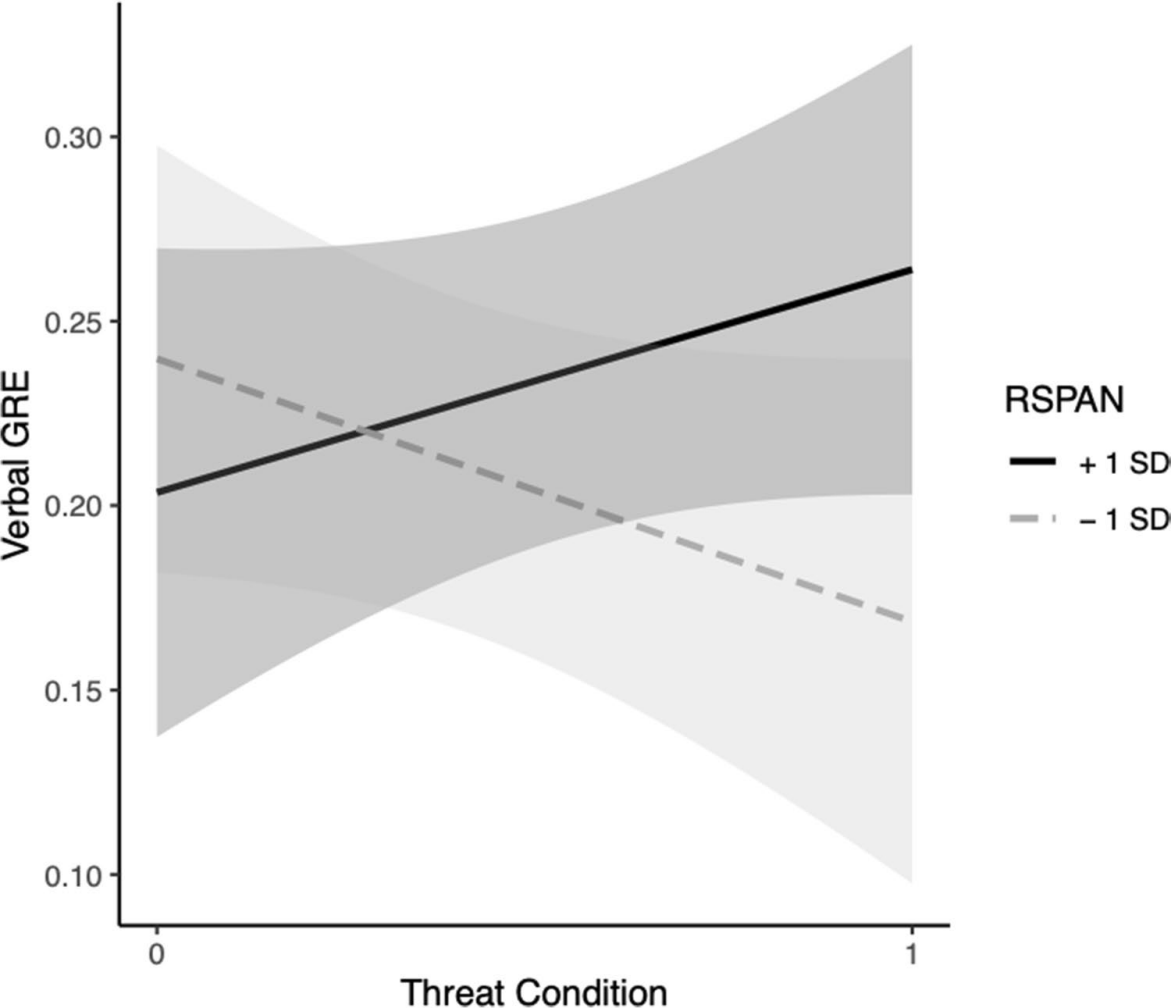
Black students’ WMC (via RSPAN) moderation model for threat on GRE-V. *Note*. Grey bands represent 95% Confidence Intervals

For the verbal GRE, in Step 1, the effects of OSPAN, *b* = 0.0032, *t*(39) = 1.31, *p* = 0.198, condition, *b* = 0.059, *t*(39) = 1.16, *p* = 0.26, and the model were nonsignificant, *F*(2, 39) = 1.45, Multiple *R*^2^ = 0.069,* p* = 0.25. In Step 2, the effect of OSPAN trended toward significance at the 0.06 level, *b* = 0.0061, *t*(38) = 1.93, *p* = 0.06, indicating that there was only an effect of trait WMC (at the 0.06 level) such that higher OSPAN scores were associated with higher predicted scores on the verbal GRE. The effects of condition, *b* = 0.031, *t*(38) = 0.582, *p* = 0.56, the interaction, *b* = − 0.0070, *t*(38) = − 1.42, *p* = 0.16, and the model, *F*(3, 38) = 1.67, Multiple *R*^2^ = 0.116, were not significant (see Fig. 4).

**Fig. 5:**
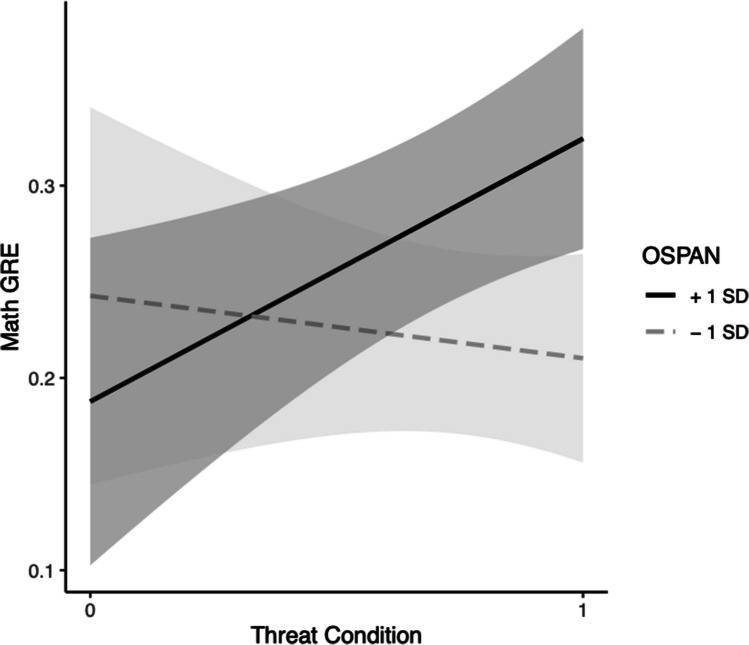
Black students’ WMC (via OSPAN) moderation model for threat on GRE-M. *Note*. Grey bands represent 95% confidence intervals

For the math GRE, in Step 1, the effects of OSPAN, *b* = 0.0032, *t*(39) = 1.08, *p* = 0.29, condition, *b* = 0.017, *t*(39) = 0.27, *p* = 0.79, and the model were nonsignificant, *F*(2, 39) = 0.61, Multiple *R*^2^ = 0.030,* p* = 0.55. In Step 2, none the effects of OSPAN, *b* = 0.0059, *t*(38) = 1.5, *p* = 0.14, condition, *b* = − 0.0088, *t*(38) = − 0.13, *p* = 0.89, their interaction, *b* = *− *0.0065, *t*(38) = − 1.08, *p* = 0.29, or the model, *F*(3, 38) = 0.80, Multiple *R*^2^ = 0.059 were significant (see Fig. 5).

### Discussion

Contrary to our predictions, we found no direct ST effects on performance, and by extension, we did not show evidence that state WMC mediates the effect of ST on standardized test performance. However, we found some evidence supporting our second hypothesis, that higher trait WMC moderated the effect of ST on standardized test performance, but only when baseline WMC was assessed using the RSPAN. Students with higher or lower trait WMC scores on the OSPAN performed similarly in terms of predicted scores on the math and verbal GREs. This is interesting because it implies that whether trait WMC moderates ST in high-achieving students depends on the kind of threat and the kind of task domain as to whether WMC is protective against ST effects. These data suggest that higher trait WMC may protect Black students from the effect of ST, but specifically for ST activated for the quantitative domain. In the quantitative capacity and intelligence threat condition, higher trait WMC predicted higher scores on the math and verbal GREs relative to lower WMC. This might also suggest that due to these greater cognitive resources, higher trait WMC students may be able to remain resilient and take on the cognitive demands of ST as a challenge depending on the how ST is activated and the performance domain.

Our sample may have higher trait WMC compared with samples of students at other universities—allowing students to remain resilient in the face of ST. Previous research suggests that higher WMC is associated with higher intelligence (Conway et al., 2003) and SDTP (Daneman & Carpenter, 1980). Experiment 1 provides potential explanations for why and how people succumb and are resilient to the effects of ST from a cognitive perspective. Because ST is viewed as a social-affective environmental phenomenon that disrupts cognitive performance, our data highlight the potential benefit of having more cognitive resources at baseline in order to combat its deleterious effects.

## Experiment 2

Experiment 2 implemented the same experimental design in a new sample to further examine the role of WMC during ST for race/ethnicity. We wondered whether Experiment 1 found an absence of a ST effect due to the private university sample being more motivated and/or experienced with high-stakes testing situations, and in turn, better able to use cognitive resources of WMC to perform competitively in spite of ST. Based on recent admissions and enrollment data,^11^ possible differences in preparation, experience, and motivation in the Experiment 1 sample are especially relevant when considering boundary conditions and the effect of ST. Experiment 2 recruited highly motivated students from a large state university, where overall GPA and previous standardized test scores were slightly less competitive.^12^

The hypotheses for Experiment 2 are the same as those for Experiment 1.

### Method

#### Participants

A total of 166 undergraduates from a public state university were recruited. Participants were invited if they were at least 18 years of age,^13^ native English speakers, and self-identified as White (98 students, 66 women) or Black (68 students, 55 women). All received credit toward a course requirement for participating.

#### Design and procedure

The same experimental design as Experiment 1 was employed. ST was tested through a series of ANOVAs, and the role of WMC was tested through mediation and moderation analyses for Black students based on math or verbal ST. Materials and procedures were generally the same as Experiment 1.^14^

### Results

We recruited participants to achieve adequate statistical power based on the same criteria outlined in Experiment 1. Again, we present two “sets” of results, one for each task order condition (see Fig. 1). As in Experiment 1, the same data preparation and analytic approaches were implemented.

### Summary statistics and correlations

Descriptive statistics revealed the measures of WMC (i.e., OSPAN and RSPAN) were strongly positively correlated with each other as well as moderately positively correlated with measures of SDTP (i.e., math and verbal GREs). These trends are reported in Table 3 below.

**Table 3:** Experiment 2 summary statistics

	1	2	3	4	5	6	7
1. ospan	--						
2. rspan	.725***	--					
3. gremProp	.174*	.189*	--				
4. grevProp	.202**	.214**	.435***	--			
5. STAI	−0.046	-.155*	0.008	−0.018	--		
6. gremAttProp	-.199**	-.193**	0.112	−0.035	−0.032	--	
7. grevAttProp	-.151*	-.218***	0.134	0.077	−0.113	0.584***	--
*M*	55.21	50.79	0.27	0.26	12.8	0.87	0.91
*SD*	14.9	14.89	0.12	0.15	3.86	0.16	0.14

The top half of the table reports Pearson’s *r *correlations and the bottom half reports *M* and *SD* for Experiment 2 measures

*ospan* Operation Span, *rspan* Reading Span, *gremProp* Proportion Correct on Math GRE, *grevProp* Proportion Correct on Verbal GRE, *gremAttProp* Proportion Attempted on Math GRE, *grevAttProp* Proportion Attempted on Verbal GRE, *STAI* Speilberger State-Trait Anxiety Inventory

***p <.001, **p <.01, *p <.05.

#### Quantitative capacity and intelligence condition

##### Baseline comparison 

An independent samples *t*-test found no significant difference in RSPAN between races, *t*(79) = − 1.17, *p* = 0.25, Cohen’s *d* = 0.26.

##### Threat effects^15^

Only the interaction effect on the second standardized test, the verbal GRE, was trending toward significance at the 0.11 level (see Table 4). Pairwise comparisons indicated that Black students scored lower on the verbal GRE but not significantly so under threat compared with the control (*M* diff = − 0.00047, *p* = 0.99). White students tended to score lower under threat compared with control (*M* diff = − 0.098, *p* = 0.10). All other interaction effects for the quantitative capacity and intelligence threat type were non-significant (see Table 4).

**Table 4:** Experiment 2 stereotype threat effects

*Experiment 2 Analysis of Variance Summary Table*										
Dependent Measure	Quantitative Capacity and Intelligence Condition				Race by Condition Interaction Effect					
	White Students		Black Students							
	Threat	Control	Threat	Control	*F*	*p*	partial *η2*	*b*	*t*	*CI *95%
	*M (SD) *(*n *= 27)	*M (SD *(*n *= 17*)*	*M (SD) *(*n *= 17)	*M (SD) *(*n *= 20)						
OSPAN	55.67 (15.58)	57.59 (16.11)	56.47 (17.13)	49.50 (16.98)	1.44	=.23	0.018	8.89	1.20	[−5.86, 23.64]
GRE-M	.31 (.12)	.33 (.13)	.23 (.09)	.19 (.06)	2.009	=.16	0.025	0.069	1.42	[-.028,.166]
GRE-V	.27 (.15)	.36 (.18)	.22 (.10)	.22 (.09)	2.51	=.11	0.032	0.098	1.58	[-.025,.22]
Dependent Measure	Verbal Capacity and Intelligence Condition				Race by Condition Interaction Effect					
	White Students		Black Students							
	Threat	Control	Threat	Control	*F*	*p*	partial *η2*	*b*	*t*	*CI *95%
	*M (SD) *(*n *= 28)	*M (SD) *(*n *= 26)	*M (SD) *(*n *= 20)	*M (SD) *(*n *= 11)						
RSPAN	54.29 (12.65)	52.22 (15.51)	44.10 (17.98)	55.55 (10.40)	3.98	=.049	.047	−13.62	−1.99	[−27.20, -.030]
GRE-V	.30 (.16)	.27 (.21)	.21 (.10)	.18 (.10)	0.022	=.88	0.00027	0.011	0.18	[-.14,.16]
GRE-M	.30 (.13)	.28 (.16)	.26 (.11)	28 (.25)	0.31	=.58	0.0038	0.037	0.55	[-.088,.15]

In the verbal capacity and intelligence condition, *p* <.0083 was used for significance in cases for Bonferroni correction

*OSPAN* Operation Span, *RSPAN* Reading Span, *GRE-M* Proportion Correct on Math GRE, *GRE-V* Proportion Correct on Verbal GRE

For the OSPAN measure, we found nonsignificant effects of condition, *F*(1, 77) = 0.37,* p* = 0.55, η_p_^2^ = 0.0048,* b* = *− *1.92, *t*(77) = − 0.38, CI_95%_ [− 12.02, 8.17], *p* > 0.05, and race, *F*(1, 77) = 1.03, *p* = 0.31, η_p_^2^ = 0.011, *b* = *− *8.09, *t*(77) = − 1.50, CI_95%_ [− 18.84, 2.67], *p* > 0.05.

The GRE-M measure found a main effect of race, *F*(1, 77) = 19.4, *p* = 0.000034, η_p_^2^ = 0.193, *b* = *− *0.14, *t*(77) = − 3.97, CI_95%_ [− 0.21, − 0.071], *p* < 0.001, indicating that White students (*M* = 0.31, *SD* = 0.13) significantly outperformed Black students (*M* = 0.21, *SD* = 0.08) on the math GRE. There was no significant effect of condition, *F*(1, 77) = 0.11, *p* = 0.74, η_p_^2^ = 0.0015, *b* = *− *0.024, *t*(77) = − 0.73, CI_95%_ [− 0.091, 0.042], *p* > 0.05.

The GRE-V measure found a significant effect of race where White students performed significantly higher (*M* = 0.30, *SD* = 0.17) on the verbal GRE compared with Black students (*M* = 0.22, *SD* = 0.10), *F*(1, 77) = 7.1, *p* = 0.0095, η_p_^2^ = 0.098, *b* = *− *0.14, *t*(77) = − 3.13, CI_95%_ [− 0.23, − 0.051], *p* < 0.01. The effect of condition was marginally significant at the 0.09 level, *F*(1, 77) = 2.90, *p* = 0.09, η_p_^2^ = 0.036, *b* = *− *0.098, *t*(77) = − 2.33, CI_95%_ [− 0.18, − 0.014], *p* < 0.05, indicating that performance was lower in the threat condition (*M* = 0.25, *SD* = 0.14) compared with the control (*M* = 0.29, *SD* = 0.15).

##### Mediation and moderation

We did not show strong evidence for a threat effect in the quantitative capacity and intelligence condition. However, we still ran the mediation analyses and refer the reader to the Supplemental Information.

Testing whether trait WMC (as measured by RSPAN) moderates the effect of ST on standardized test performance, a hierarchical regression was conducted for Black students with the math and verbal GRE.

For math GRE in Step 1, the effects of RSPAN, *b* = 0.000071, *t*(34) = − 0.085, *p* = 0.93, and the model, *F*(2, 34) = 1.45, Multiple *R*^2^ = 0.078, *p* = 0.25, were nonsignificant. The effect of condition was marginally significant at the 0.09 level, *b* = 0.045, *t*(34) = 1.7, *p* = 0.09. In Step 2, the effects of RSPAN, *b* = 0.00034, *t*(33) = 0.29, *p* = 0.77, condition, *b* = 0.043, *t*(33) = 1.59, *p* = 0.12, the interaction, *b* = *− *0.00086, *t*(33) = − 0.51, *p* = 0.62, and the model, *F*(3, 33) = 1.03, Multiple *R*^2^ = 0.086, *p* = 0.39, were nonsignificant. These findings indicate that under threat higher trait WMC did not provide a benefit compared with lower trait WMC on the math GRE.

For verbal GRE in Step 1, the effects of RSPAN, *b* = 0.00083, *t*(34) = 0.81, *p* = 0.43, condition, *b* = *− *0.0042, *t*(34) = − 0.13, *p* = 0.89 and the model, *F*(2, 34) = 0.326, Multiple *R*^2^ = 0.0188,* p* = 0.72, were nonsignificant. In Step 2, the effects of RSPAN, *b* = *− *0.0011, *t*(33) = − 0.83, *p* = 0.42, condition, *b* = 0.0052, *t*(33) = 0.165, *p* = 0.87, and the model, *F*(3, 33) = 1.67, Multiple *R*^2^ = 0.132, *p* = 0.19, were nonsignificant. The interaction of RSPAN and condition was significant, *b* = 0.0041, *t*(33) = 2.07, *p* = 0.046. Simple slopes tests indicated that under threat, higher WMC participants had higher predicted scores on the verbal GRE compared with those with lower WMC,* b* = 0.0030, *p* = 0.045. There was no significant change in predicted GRE scores in the control condition, *b* = − 0.0011, *p* = 0.42. Based on the significant interaction, the models in Steps 1 and 2 were tested for a significant change in their variances. Analysis of change in model variances indicated there was a significant difference, *F*(1, 33) = 4.3, *p* = 0.046, providing further evidence for moderation of trait WMC on ST for Black students’ verbal GRE scores (see Fig. 4).

#### Verbal capacity and intelligence condition

##### Baseline comparison

Independent-samples *t* test found that although White students performed about 4 points higher on average OSPAN, this difference was not significant,^16^*t*(83) = − 1.38, *p* = 0.17, Cohen’s *d* = 0.31.

##### Threat effects^17^

 Only a significant race by condition interaction was found on WMC via the RSPAN task (see Table 4). Follow-up tests revealed White students in the threat condition did not differ from White students in the control (*M* diff = 2.17, *p* = 0.95). In contrast, Black students under threat tended to experience a performance decrease on RSPAN relative to Black students in the control condition; however, this finding was not significant (*M* diff = − 11.4, *p* = 0.17). All other interaction effects were nonsignificant (see Table 4).

For main effects on the RSPAN, the effects of condition, *F*(1, 81) = 0.61, *p* = 0.44, η_p_^2^ = 0.0074, *b* = 2.17, *t*(81) = 0.54, CI_95%_ [− 5.81, 10.15], *p* > 0.05, and race, *F*(1, 81) = 2.34, *p* = 0.13, η_p_^2^ = 0.024, *b* = 3.43, *t*(81) = 0.64, CI_95%_ [− 7.11, 13.97], *p* > 0.05, were nonsignificant.

For GRE-V, Levene’s test revealed a significant homogeneity of variance violation, *F*(3, 81) = 3.1, *p* = 0.031. A Bonferroni correction was used to compute the new significance criteria of 0.0083. Results indicated only a main effect of race, *F*(1, 81) = 5.26, *p* = 0.024, η_p_^2^ = 0.065, *b* = *− *0.093, *t*(81) = − 1.62, CI_95%_ [− 0.21, 0.021], *p* = 0.11; however, based on the Bonferroni criteria of 0.0083, this effect was nonsignificant. The effect of condition was also nonsignificant, *F*(1, 81) = 0.67, *p* = 0.42, η_p_^2^ = 0.0082, *b* = 0.025, *t*(81) = 0.58, CI_95%_ [− 0.061, 0.11], *p* > 0.05.

For math, GRE^18^ results indicated the effect of race was nonsignificant, *F*(1, 80) = 2.5, *p* = 0.12, η_p_^2^ = 0.035, *b* = *− *0.071, *t*(80) = − 1.49, CI_95%_ [− 0.17, 0.024], *p* > 0.05. The effect of condition was also nonsignificant, *F*(1, 80) = 1.06, *p* = 0.31, η_p_^2^ = 0.013, *b* = 0.018, *t*(80) = 0.52, CI_95%_ [− 0.051, 0.088], *p* > 0.05.

##### Mediation and moderation

Although we found a significant threat effect on the RSPAN task, we did not show strong evidence for a threat effect on SDTP in the verbal capacity and intelligence condition. We still ran the analyses and refer the reader to the Supplemental Information.

Testing whether trait WMC (as measured by OSPAN) moderates the effect of ST on standardized test performance, a hierarchical regression was conducted for Black students with the outcomes of math and verbal GRE.

In Step 1, looking at the verbal GRE the effects of condition, *b* = 0.034, *t*(27) = 0.82, *p* = 0.42, OSPAN, *b* = *− *0.00011, *t*(27) = − 0.087, *p* = 0.93, and the model, *F*(2, 27) = 0.34, Multiple *R*^2^ = 0.025,* p* = 0.71, were nonsignificant.

In Step 2, predicting verbal GRE, the effect of OSPAN, *b* = *− *0.0015, *t*(26) = − 0.55, *p* = 0.59, condition, *b* = 0.035, *t*(26) = 0.84, *p* = 0.41, the interaction, *b* = 0.0018, *t*(26) = 0.57, *p* = 0.57, and the model, *F*(3, 26) = 0.33, Multiple *R*^2^ = 0.037, *p* = 0.80, were not significant. Taken together, these results reveal a lack of support for trait WMC on the OSPAN moderating the effect of threat on verbal GRE.

In Step 1 for math GRE, the effect of condition, *b* = 0.059, *t*(27) = 1.6, *p* = 0.12, was nonsignificant, the effect of OSPAN, *b* = 0.0024, *t*(27) = 2.18, *p* = 0.039, and the model, *F*(2, 27) = 3.4, Multiple *R*^2^ = 0.201,* p* = 0.049, were significant. These results indicate that higher scores on the OSPAN predicted higher scores on the math GRE.

In Step 2, predicting math GRE, the effect of OSPAN, *b* = *− *0.0018, *t*(26) = − 0.79, *p* = 0.43, was nonsignificant. The effect of condition, *b* = 0.064, *t*(26) = 1.9, *p* = 0.076, approached significance, indicating that the threat effect predicted higher scores on the math GRE. The interaction, *b* = 0.0054, *t*(26) = 2.15, *p* = 0.041, and the model, *F*(3, 26) = 4.10, Multiple *R*^2^ = 0.32, *p* = 0.017, were significant. Simple slopes tests indicated that under threat, higher WMC participants had higher predicted scores on the math GRE compared with those with lower WMC,* b* = 0.0036, *p* = 0.0051. There was no significant change in predicted GRE scores in the control condition, *b* = − 0.0018, *p* = 0.43 (see Fig. 9). Based on the significant interaction term, an ANOVA was conducted in order to determine whether there was a significant difference in the variance accounted for between these models. Results revealed a significant difference in the variance, *F*(1, 26) = 4.62, *p* = 0.041, providing additional support for moderation (see Fig. 5).

### Discussion

There was limited evidence for ST effects impacting performance on outcome measures of WMC and standardized test performance. Only in the case of ST for verbal capacity and intelligence on the RSPAN did these data reveal evidence of a threat effect. Overall, students appeared to be resilient to the effects of ST. Here, it was expected that because the student sample came from a less “selective”^19^ population of undergraduate students, ST effects would be found in addition to evidence that ST is moderated by high trait WMC and mediated by state WMC. Instead, there was no evidence to support the notion that state WMC mediates ST—in most cases there was no threat effect revealed. Although, there was a pattern of some evidence supporting our second hypothesis that trait WMC moderates the effect of ST on both math and verbal SDTP providing a performance benefit for Black students. We believe this could be the case because having higher WMC span corresponds with more domain-general resources, which is less limiting for higher WMC span (see Kovacs et al., 2019; Kovacs & Conway, 2016). Moreover, these greater domain-general resources means that performance is less impacted for higher WMC span than lower WMC span students when subjected to the cognitive demands of identity-threatening situations like racial/ethnic ST effects. Moreover, like in Experiment 1, higher trait WMC students may be able to remain resilient and take on ST as a challenge depending on how ST is activated and the performance domain. In Experiment 2 however, our students showed resilience to ST effects on the second GRE task—when the performance domain and domain of ST were different (see Figs. 1, 4, and 5).

### Combined experiments analyses

Due to time and resource constraints impacting data collection we combined data from Experiments 1 and 2 in order to provide a more powerful test of our hypotheses. In addition to this, we ran Bayesian regression in an effort to provide a more complete picture about the strength of the evidence from these data. We present the combined samples analysis results below.

### Quantitative capacity and intelligence condition

#### Baseline comparison

An independent-samples *t* test found a significant difference in RSPAN between racial groups, *t*(279) = − 3.15, *p* = 0.0018, Cohen’s *d* = 0.41, with White students (*M* = 58.10, *SD* = 12.40) performing higher than Black students (*M* = 52.68, *SD* = 14.63).

#### Threat effects^20^

 We next examined the effects of the Race × Condition interaction for the combined samples. For performance on the OSPAN, the effect of race was significant, *F*(1, 277) = 8.62, *p* = 0.0036, η_p_^2^ = 0.031. The effects of condition, *F*(1, 277) = 0.222, *p* = 0.64, η_p_^2^ = 0.0008, and the interaction, *F*(1, 277) = 1.31, *p* = 0.25, η_p_^2^ = 0.0047 were nonsignificant.

Examining performance on the math GRE, the effect of race was significant, *F*(1, 277) = 43.35, *p* < 0.0001, η_p_^2^ = 0.134. The effects of condition, *F*(1, 277) = 0.079, *p* = 0.78, η_p_^2^ = 0.00029, and the interaction, *F*(1, 277) = 2.51, *p* = 0.11, η_p_^2^ = 0.0090, were nonsignificant.

Turning to performance on the verbal GRE, a violation of homogeneity was detected thus we used the Bonferroni correction of 0.0083. Based on this, only the effect of race was significant, *F*(1, 277) = 34.23, *p* < 0.0001, η_p_^2^ = 0.112. The effects of condition, *F*(1, 277) = 0.999, *p* = 0.32, η_p_^2^ = 0.0036, and the interaction, *F*(1, 277) = 1.85, *p* = 0.18, η_p_^2^ = 0.0066, were nonsignificant.

#### Mediation and moderation 

We tested for mediation with state WMC in the combined samples analysis and refer the reader to Supplemental Information for those results. Testing whether trait WMC (as measured by RSPAN) moderates the effect of ST on standardized test performance, a hierarchical regression was conducted for Black students, with the outcomes of math and verbal GRE.

In Step 1, looking at the math GRE, the effect of RSPAN, *b* = 0.0037, *t*(79) = 2.75, *p* = 0.0074, was significant. The effect of condition, *b* = 0.049, *t*(79) = 1.26, *p* = 0.0074, was nonsignificant, but the model, *F*(2, 79) = 5.21, Multiple *R*^2^ = 0.117,* p* < 0.01, was significant.

**Fig. 6:**
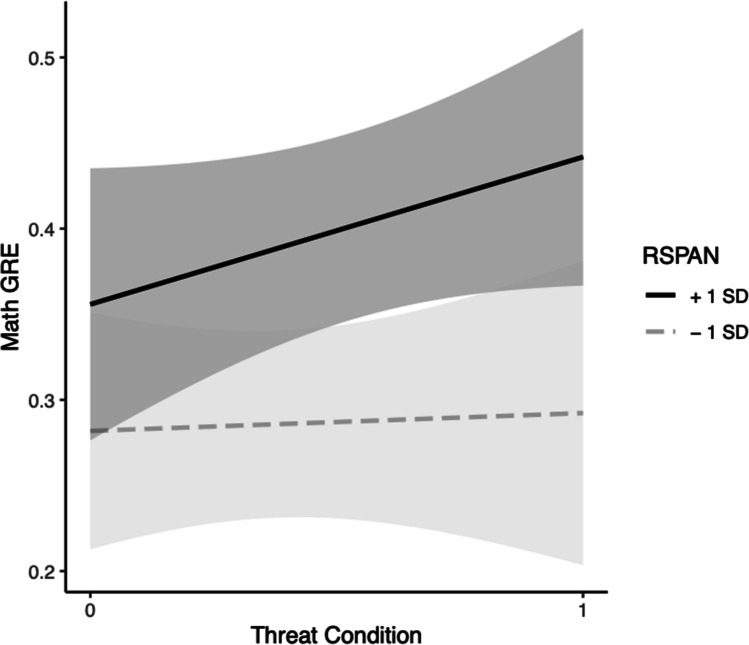
Black students’ WMC (via RSPAN) moderation model for threat on GRE-M. *Note*. Grey bands represent 95% confidence intervals

In Step 2, predicting math GRE, the effect of RSPAN, *b* = 0.0025, *t*(78) = 1.39, *p* = 0.17, condition, *b* = 0.058, *t*(78) = 1.44, *p* = 0.16, the interaction, *b* = 0.0026, *t*(78) = 0.957, *p* = 0.34, were nonsignificant. The model, *F*(3, 78) = 3.78, Multiple *R*^2^ = 0.127, *p* = 0.014, was significant. We also tested the change in model variances and simple slopes. Although there was not a significant change in the model variances accounted for *F*(1, 78) = 0.92, *p* = 0.34, under threat, higher WMC participants had higher predicted scores on the math GRE compared with those with lower WMC, *b* = 0.0051, *p* = 0.013; however, there was no significant difference for those in the control condition, *b* = 0.0025, *p* = 0.17. Taken together, these results reveal weak support for trait WMC on the RSPAN moderating the effect of threat on the math GRE (see Fig. 6).

In Step 1, looking at the verbal GRE the effect of RSPAN, *b* = 0.0039, *t*(79) = 2.64, *p* = 0.0099, was significant. The effect of condition, *b* = 0.013, *t*(79) = 0.29, *p* = 0.77, was not significant, but the model, *F*(2, 79) = 3.73, Multiple *R*^2^ = 0.086,* p* = 0.028, was significant.

**Fig. 7:**
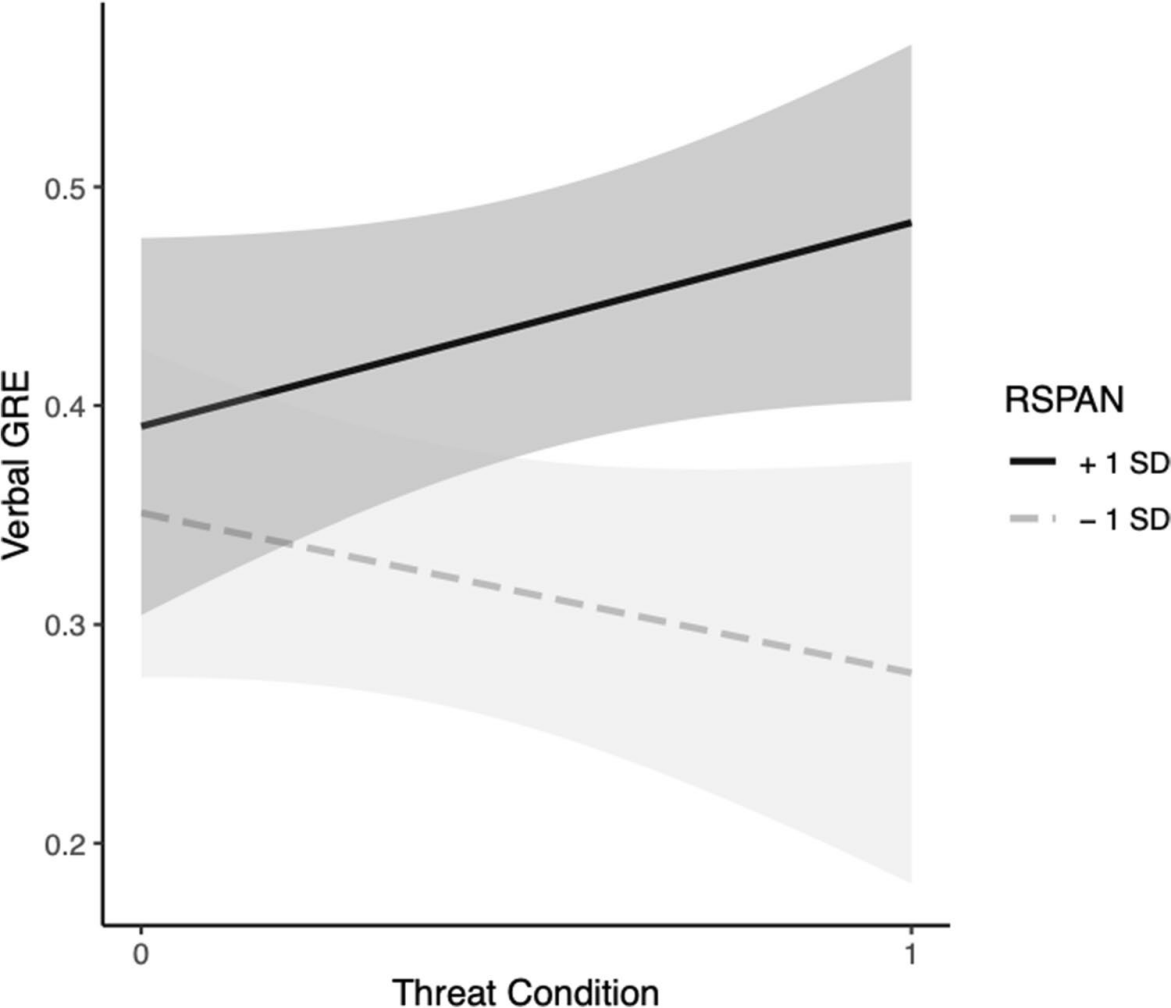
Black students’ WMC (via RSPAN) moderation model for threat on GRE-V. *Note*. Grey bands represent 95% confidence intervals

In Step 2, predicting verbal GRE, the effect of RSPAN, *b* = 0.0014, *t*(78) = 0.69, *p* = 0.049, was significant, however, condition, *b* = 0.031, *t*(78) = 0.71, *p* = 0.48, was nonsignificant. The interaction, *b* = 0.0057, *t*(78) = 1.94, *p* = 0.056, was marginal at the 0.056 level and the model, *F*(3, 78) = 3.83, Multiple *R*^2^ = 0.1283, *p* = 0.013, was significant. We also tested the change in model variance, which was also marginally significant at the 0.056 level, *F*(1, 78) = 3.76, *p* = 0.056. To further unpack this, we examined the simple slope analysis revealing that under threat higher WMC participants has higher predicted scores on the verbal GRE compared with those with lower WMC, *b* = 0.0070, *p* = 0.0018. There was no significant change in predicted GRE scores in the control condition *b* = 0.0014, *p* = 0.49. Taken together, these results also reveal weak support for trait WMC on the RSPAN moderating the effect of threat on the verbal GRE (see Fig. 7).

#### Bayesian regression analyses

To better understand the strength of evidence supporting our hypotheses we ran Bayesian regressions for both mediation and moderation analyses for working memory and ST effects. Despite the lack of a consistent race by threat interaction, we ran as planned the Bayesian mediation analyses and include those in supplemental information.

We used the BayesFactor package in R (Morey & Rouder, 2015) to compute Bayes factors for main-effects-only models and the main effects plus the interaction terms models. Each of these was run compared with a null or intercept only model. To test for moderation, we computed the Bayes factors for the main-effects-only model and compared those to the main effects plus interaction term models. We include the full results of the Bayesian regression moderation analyses for the quantitative capacity condition in Table 5 and discuss the relevant results of these analyses in more detail below.

**Table 5:** Combined samples Bayesian regression quantitative capacity condition

Bayesian Multiple Regression Moderation Summary Table		
Predictor(s)	Quantitative Capacity and Intelligence Condition	
	Dependent Measure	
	OSPAN	Model Comparison
	*Bayes Factor*	*Bayes Factor*
*Main Effects*		
RSPAN	51453732354	
Condition	.27	
*Main Effects *+ *Interaction*		
RSPAN	51453732354	
Condition	.27	
RSPAN+ Condition	7740505205	
RSPAN+Condtion+ RSPAN*Condition	1966209621	3.936765
Predictor(s)	Dependent Measure	
	Math GRE	Model Comparison
	*Bayes Factor*	*Bayes Factor*
*Main Effects*		
RSPAN	9.42	
Condition	.72	
*Main Effects *+ *Interaction*		
RSPAN	9.42	
Condition	.72	
RSPAN+Condition	5.21	
RSPAN+Condition+ RSPAN*Condition	2.57	2.028387 (inverse, 0.4930027)
	Dependent Measure	
	Verbal GRE	Model Comparison
Predictor(s)	*Bayes Factor*	*Bayes Factor*
*Main Effects*		
RSPAN	5.48	
Condition	.28	
*Main Effects *+ *Interaction*		
RSPAN	5.48	
Condition	.28	
RSPAN+Condition	1.62	
RSPAN+Condition+ RSPAN*Condition	2.72	.5982358 (inverse, 1.671582)

Main effects represent models with both predictors entered together. Main effects plus interaction represent models with predictors and their interaction entered simultaneously. Model comparison indicates a comparison of the main-effects-only model with the main effects plus interaction model. Inverse indicates the inverse of Bayes factors in the comparison. OSPAN = Operation Span, RSPAN = Reading Span, Condition = Stereotype Threat Effect.

In the main-effects-only model, the Bayes factor (*BF* = 51453732354) indicated there was very strong evidence against the null in the model of RSPAN predicting performance on the OSPAN. For the main effect of threat condition predicting OSPAN the Bayes factor was less than 1 (*BF* = 0.27) indicating evidence in support of the null model. In the model comparison the Bayes factor (*BF* = 3.94) indicated strong evidence in favor of the main-effects-only model.

When looking at the effects on the math GRE the Bayes factor (*BF* = 9.42) for the main effect of RSPAN only revealed strong evidence against the null. The Bayes factor for the main effect of threat condition contained a Bayes factor less than 1 (*BF* = 0.72), indicating evidence in favor of the null. In the model comparison of main-effects-only to main effects plus interaction, the Bayes factor (*BF* = 2.03) revealed anecdotal evidence in favor of the main-effects-only model.

When looking at the effects on the verbal GRE the Bayes factor (*BF* = 5.48) for the main effect of RSPAN revealed strong evidence against the null. The Bayes factor for the main effect of threat condition contained a Bayes factor less than 1 indicating (*BF* = 0.28) evidence in support of the null. In the main effects plus the interaction model predicting the verbal GRE the model comparison Bayes factor (*BF* = 1.67) revealed anecdotal evidence in favor of the interaction model compared with the main-effects-only model.

### Verbal capacity and intelligence condition

#### Baseline comparison

An independent-samples *t* test found a significant difference in OSPAN between racial/ethnic groups, *t*(334) = − 2.56, *p* = 0.011, Cohen’s *d* = 0.34. with White students (*M* = 61.25, *SD* = 11.88) performing significantly higher than Black students (*M* = 57.13, *SD* = 12.66).

#### Threat effects^21^

 We next examined the effects of the Race × Condition interaction for the combined samples. For performance on the RSPAN, the effect of race was significant, *F*(1, 332) = 13.97, *p* = 0.00022, η_p_^2^ = 0.040. The effects of condition, *F*(1, 332) = 0.001, *p* = 0.97, η_p_^2^ = 0.000004, and the interaction, *F*(1, 332) = 1.94, *p* = 0.16, η_p_^2^ = 0.0058, were nonsignificant.

Examining performance on the verbal GRE, the effect of race was significant, *F*(1, 332) = 32.78, *p* < 0.0001, η_p_^2^ = 0.085. The effects of condition, *F*(1, 332) = 1.46, *p* = 0.23, η_p_^2^ = 0.00044, and the interaction, *F*(1, 332) = 0.28, *p* = 0.59, η_p_^2^ = 0.00084, were nonsignificant.

Turning to performance on the math GRE, the effect of race was significant, *F*(1, 331) = 30.20, *p* < 0.0001, η_p_^2^ = 0.083. The effects of condition, *F*(1, 332) = 0.005, *p* = 0.94, η_p_^2^ = 0.000016, and the interaction, *F*(1, 332) = 0.005, *p* = 0.95, η_p_^2^ = 0.000014, were nonsignificant.

#### Mediation and moderation

Again, there was no evidence for a threat effect in the combined samples for mediation; however, we ran them as planned and report results in the Supplemental Information.

Testing whether trait WMC (as measured by OSPAN) moderates the effect of ST on standardized test performance, a hierarchical regression was conducted for Black students with the outcomes of math and verbal GRE.

In Step 1, looking at the verbal GRE the effect of OSPAN, *b* = 0.0042, *t*(68) = 2.17, *p* = 0.033, was significant. The effect of condition, *b* = 0.0028, *t*(68) = 0.058, *p* = 0.95, was nonsignificant. The model, *F*(2, 68) = 3.73, Multiple *R*^2^ = 0.065,* p* = 0.10, was nonsignificant.

In Step 2, predicting verbal GRE, the effect of OSPAN, *b* = 0.0053, *t*(67) = 1.73, *p* = 0.088, condition, *b* = *− *0.0035, *t*(67) = − 0.069, *p* = 0.95, were not significant. The interaction, *b* = *− *0.0019, *t*(67) = − 0.49, *p* = 0.63, and the model, *F*(3, 76) = 1.63, Multiple *R*^2^ = 0.068, *p* = 0.019, were also not significant. Taken together, these results do not provide support for trait WMC on the OSPAN moderating the effect of race-related threat on verbal GRE.

In Step 1, looking at the math GRE the effect of OSPAN, *b* = 0.0040, *t*(68) = 11.28, *p* = 0.031, was significant. The effect of condition, *b* = 0.0062, *t*(68) = 0.13, *p* = 0.89, and the model, *F*(2, 68) = 2.43, Multiple *R*^2^ = 0.067,* p* = 0.095, were not significant.

In Step 2 predicting math GRE, the effect of OSPAN, *b* = 0.0048, *t*(67) = 1.65, *p* = 0.10, condition, *b* = 0.0019, *t*(67) = 0.039, *p* = 0.97, were not significant. The interaction, *b* = *− *0.0013, *t*(67) = − 0.36, *p* = 0.72, and the model, *F*(3, 67) = 1.64, Multiple *R*^2^ = 0.069, *p* = 0.19, were not significant. These results also do not provide support for trait WMC on the OSPAN moderating the effect of race-related threat on the math GRE.

#### Bayesian regression analyses 

Again, due to the lack of a consistent Race × Threat interaction, we still ran the Bayesian mediation analyses as planned and include those in the Supplemental Information.

We used the Bayes Factor package in R (Morey & Rouder, 2015) to compute Bayes Factors for main-effects-only models and the main effects plus the interaction terms models. Each of these was run compared to a null or intercept only model. To test for moderation, we computed the Bayes Factors for the main-effects-only model and compared those to the main effects plus interaction term models. We include the full results of the Bayesian regression moderation analyses for the verbal capacity condition in Table 6 and discuss the results in greater detail below.

**Table 6:** Combined samples Bayesian regression verbal capacity condition

Bayesian Multiple Regression Moderation Summary Table		
Predictor(s)	Verbal Capacity and Intelligence Condition	
	Dependent Measure	
	RSPAN	Model Comparison
	*Bayes Factor*	*Bayes Factor*
*Main Effects*		
OSPAN	626854.1	
Condition	.46	
*Main Effects + Interaction*		
OSPAN	626854.1	
Condition	.46	
OSPAN+ Condition	189054.3	
OSPAN+Condtion+ OSPAN*Condition	66060.4	2.86184
Predictor(s)	Dependent Measure	
	Verbal GRE	Model Comparison
	*Bayes Factor*	*Bayes Factor*
*Main Effects*		
OSPAN	1.84	
Condition	.25	
*Main Effects *+ *Interaction*		
OSPAN	1.84	
Condition	.25	
OSPAN+Condition	.59	
OSPAN+Condition+ OSPAN*Condition	.25	2.344554
Dependent Measure		
Math GRE	Model Comparison	
Predictor(s)	*Bayes Factor*	*Bayes Factor*
*Main Effects*		
OSPAN	1.94	
Condition	.24	
*Main Effects *+ *Interaction*		
OSPAN	1.94	
Condition	.25	
OSPAN+Condition	.62	
OSPAN+Condition+ OSPAN*Condition	.25	2.461715

Main effects represent models with both predictors entered together. Main effects plus interaction represent models with predictors and their interaction entered simultaneously. Model comparison indicates a comparison of the main-effects-only model with the main effects plus interaction model. Inverse indicates the inverse of Bayes factors in the comparison

*OSPAN* Operation Span, *RSPAN* Reading Span, *Condition* Stereotype Threat Effect.

In the main-effects-only model predicting state working memory capacity on the RSPAN, the Bayes factor (*BF* = 626,854.1) for OSPAN indicated strong evidence against the null. The Bayes factor for the effect of threat only was less than 1 (*BF* = 0.46) indicating support for the null. In the main effects plus interaction model predicting RSPAN the model comparison Bayes factor (*BF* = 2.86) indicated anecdotal evidence in favor of the main-effects-only model.

In the main-effects-only model predicting the verbal GRE, the Bayes factor (*BF* = 1.84) for the OSPAN revealed anecdotal evidence against the null. The threat effect contained a Bayes factor less than 1 indicating (*BF* = 0.25) evidence in support of the null. In the main effects plus interaction model predicting verbal GRE the model comparison Bayes factor (*BF* = 2.34) revealed anecdotal evidence in favor of the main-effects-only model.

For the main-effects-only model, predicting math GRE the Bayes factor (*BF* = 1.94) for the OSPAN revealed anecdotal evidence against the null. The Bayes factor for the main effect of threat condition contained a Bayes factor less than 1 (*BF* = 0.24), indicating support for the null. In the main effects plus interaction model, predicting math GRE the model comparison Bayes factor (*BF* = 2.46) revealed anecdotal evidence in favor of the main-effects-only model.

### Discussion

Taken together, the combined samples analysis provides additional support for our second hypothesis that higher trait WMC aids standardized test performance for racial/ethnic minority students who are faced with identity-threatening situations. These results suggest that having greater domain-general resources in WMC could be a protective factor for students experiencing forms of ST, such that they remain resilient to ST during standardized testing situations; however, there may also be differences in performance based on quantitative and verbal domains.

## General discussion

The goal of the present work was to investigate WMC as a cognitive factor in relation to performance under ST. We explored WMC as both state and trait variables as a mediator and/or moderator of ST for race/ethnicity. Although WMC has been previously explored as a state and trait variable (Ilkowska & Engle, 2010) and previous research has suggested the importance of considering individual differences in WMC with regard to ST (Regner et al., 2010; Schmader & Johns, 2003; Schmader et al., 2008), to our knowledge, exploring ST with both state and trait measures of WMC is novel and has not been investigated for ST for race/ethnicity.

Based on the results of two experiments, we found that students appeared to be resilient to the effect of ST on standardized test performance—the ST manipulation did not consistently produce a performance decrement for Black students. We found evidence of ST only in Experiment 2 for the verbal capacity and intelligence condition and only on the state WMC reading span (RSPAN) measure. Because participants appeared to be resilient to ST, we wondered whether this was due to differences in these students’ prior experience with standardized tests and beliefs about their abilities. Experiment 1 contained an especially highly motivated, high achieving student sample, so we wondered whether there was an overarching implicit belief among these students about high ability that attenuated the effect of ST on performance. This was suspected post hoc based on the method of how ST was induced—stating that the task was “highly correlated with measures of intelligence,” could activate a broader identity among these participants (see Brannon et al., 2015, for more on the shifting of the self-schema in different contexts; also, Logel et al., 2009, for shifting self-schema during ST). For example, the identity of being a student at a selective private university could protect students from feeling threatened and incidentally provide performance enhancement rather than decrement. However, based on similar patterns in Experiment 2, this did not seem as plausible but was worth mentioning here, as ST theory asserts the importance of domain identification (see Steele, 1997). In terms of resilience and motivation, previous research demonstrated that higher trait resilience as measured by grit has been shown to be moderately but not significantly associated with higher WMC (Dale et al., 2018). At present, these data are unable to disentangle whether differences in students’ beliefs about ability impact WMC or ST and performance, but future work should investigate this further.

Experiments 1 and 2 both revealed evidence that higher trait WMC moderated the effect of ST on SDTP on both the verbal and math GREs for Black students. In Experiment 1, however, moderation based on higher trait WMC was found only when students were under ST for quantitative capacity and intelligence. There was no evidence that the effect of ST for verbal capacity and intelligence on SDTP was moderated by higher trait WMC. Experiment 2 revealed that when students were threatened for intelligence and math or verbal capacities, they were able to kick performance up a notch but only on the second GRE (i.e., the GRE that did not match the domain of threat). This finding provides additional support that higher trait WMC protects Black students from ST on standardized test performance. Here, we found that the kind of threat and the domain of threat also matters.

Another perspective on these results is that having higher trait WMC resources could allow participants experiencing ST to respond by taking on the standardized test as a challenge. Some previous work suggests that racial minority students are able to perform well on standardized tests when they are viewed or characterized as a challenge (Steele & Aronson, 1995). However, if students have more cognitive resources in WMC, they might differ in the ways they focus those resources and plan to complete the task at hand. There is previous research on individual differences in WMC that shows higher and lower span individuals differ in their strategies for approaching, performing, and solving different cognitive tasks (see Ilkowska & Engle, 2010; Shipstead et al., 2016; Unsworth et al., 2013; also see Delaney & Sahakyan, 2007). However, our results suggest the ability to do this in the context of ST could also differ depending on the whether the test is in the quantitative or verbal domains. Additional research is needed to better understand individual differences in WMC and how students respond to racial/ethnic ST in different task domains.

Based on the findings of Experiments 1 and 2, it is important to note that the performance gap between White students and Black students was not removed. Instead, higher trait WMC helped Black students combat ST. Results from the combined analysis from both experiments as well as Bayesian regression analyses provided some additional support for trait WMC moderation. These results could have important implications for Black and racial/ethnic minority students and their performance on assessments that can influence admittance to higher education institutions. Additionally, these results may provide clarification for why null effects of ST have been observed in some cases—additional cognitive resources from high to extremely high trait WMC provides an advantage for combatting ST. For these reasons, WMC has been demonstrated to be an important cognitive tool for ensuring that racial minority students perform their best, particularly in high-stakes testing situations.

Another consideration is about how we found more cases of moderation when baseline WMC was on the RSPAN. This suggests that the predictive power of trait WMC using OSPAN or RSPAN may bring dissociations in the effects observed. In fact, previous research has found that because the OSPAN and RSPAN have different processing components (i.e., solving math problems for accuracy in OSPAN and reading sentences for accuracy in RSPAN), could cause the tasks to dissociate in their consistency for predicting performance on different outcomes (see Chow et al., 2016; Holden et al., 2020; Macnamara et al., 2011; Oberauer, 2009). However, this interpretation is speculative but worth further exploration.

## Limitations and future research

Overall, across two experiments, we only found ST in Experiment 2 on the state WMC RSPAN measure—highlighting replication issues. It was unclear whether replication issues were due to the language used in our manipulation (based on Schmader & Johns, 2003, stating “this task is a measure of quantitative/verbal capacity and is highly related to measures of intelligence”) or based on issues with sample size.

We aimed to recruit adequate sample sizes, but due to reported replicability issues of ST (Schimmack, 2016, 2017; also Stricker & Ward, 2004), and potential effect-size inflation due to publication bias (see Flore & Wicherts, 2014), the sample size needed is much higher than originally anticipated (unknown to us at the time of planning the current study). For these reasons, we recommend recruiting samples that *exceed* 80% power based on effect sizes reported in the literature. To help clarify replication issues, we also recommend that future investigations use more direct language as in the manipulation based on the Steele and Aronson (1995) study (i.e., “this task is diagnostic/non-diagnostic of ability”).

Another potential limitation was possible differences in cognitive or WM load across test items in the verbal and quantitative test sections. The sample GRE problems were drawn from sample study materials provided by the Educational Testing Service. These sections include a range of items that have been pretested and include percentiles of performance difficulties; however, they do not include information regarding the complexity of the items, which might correspond with how we would view as “more or less cognitively demanding items.”

The verbal section largely includes vocabulary (i.e., text completion and sentence equivalence) and reading comprehension items. Based on previous research the reading comprehension items have been linked with working memory and fluid intelligence and thus are likely to be more cognitively demanding than the text completion and sentence equivalent items (Daneman & Carpenter, 1980; De Jonge & De Jong, 1996; also see Vernucci et al., 2021). The quantitative reasoning section includes arithmetic, algebra, geometry, and data analysis problems. Previous research suggests that geometry may be the most complex or cognitively demanding among these question types, and differences in gender threat have been shown (see Huguet & Regner, 2007).

In general, more complex questions which involve more steps in order to arrive at a solution are also more cognitively demanding, so there is probably more to consider for understanding the cognitive load among the items and question domains (see Beilock & Carr, 2005; Beilock & DeCaro, 2007). For both the verbal and quantitative test sections several steps were involved in arriving at correct answers, and some questions had multiple correct answers, making it even more difficult to know which might be more cognitively demanding than others. Although this is a very interesting point, it is difficult to answer and remains outside of the scope of the present work but could be investigated more in future work.

## Conclusion

Our current work emphasizes the need to explore and challenge a fundamental assumption of universality of human information processing by factoring in the racial sociocultural context of individuals performing cognitive tasks related to academic achievement (Holden et al., 2023; Thomas et al., 2023). Although inequality and gaps in education and achievement remain, the current work helps provide a better understanding of individual differences in cognitive resources that are available for performance on competitive standardized tests. We employed a comprehensive approach, combining experimental and differential methods for investigating ST—helping the field continue to uncover how, when, and why ST operates and how to better combat it. Based on our results, future work should focus on ways to conserve precious mental resources, especially for minority students. As recent work finds that WMC is enhanced through training in general (see Jaeggi et al., 2008, 2011, 2013; Redick et al., 2013) and in more diverse and at-risk samples (see Wong et al., 2024), future work should consider additional forms of cognitive intervention that are helpful for students who are vulnerable to ST for race/ethnicity. For example, mindfulness practices might be an important avenue for future research related to ST as these practices as forms of self-regulation have been shown to improve cognitive functioning, WMC, and potentially improve SDTP (Morrison & Jha, 2015; Mrazek et al., 2013).

## Supplementary Information

Below is the link to the electronic supplementary material.

Supplementary file1 (DOCX 312 KB)

## Data Availability

The supplemental methodology information and datasets analyzed during the current study are available from the corresponding author on reasonable request.

## Footnotes

1 In the current work, we consider individual differences in WMC, also examining racial/ethnic ST effects on standardized test performance using free practice materials of verbal and quantitative reasoning sections of the GRE from the Educational Testing Service (ETS). The verbal reasoning section is described by ETS (2018) as measuring the ability to analyze and evaluate written material and synthesize information, also measuring abilities to analyze relationships among component parts of sentences and recognize relationships among words and concepts. This section contains several submeasures, including reading comprehension, text completion, and sentence equivalence questions. The quantitative reasoning section measures basic math skills, understanding of elementary math concepts, ability to reason quantitatively, and model and solve problems with quantitative methods (ETS, 2018). The section includes submeasures of arithmetic, algebra, geometry, data analysis which could involve quantitative comparisons and numeric entry questions.

2 Focusing on the role of WMC in race ST effects, our manipulation is based on the methods of Schmader and Johns (2003), and as such, we did not implement the typical “diagnostic versus nondiagnostic” manipulation as in Steele and Aronson (1995).

3 We manipulated the WMC span order and separated each set in our analyses by this factor because it corresponds with receiving different task orders (see Fig. 1 B).

4 At the time of planning this study, to our knowledge, no previous study had examined racial/ethnic ST effects with WMC as a moderator and mediator with Black students. Considering this, we planned our sample size based on the closest studies of Schmader and Johns (2003) and Regner et al. (2010). Due to limited previous research and limited information provided on effect sizes and descriptive statistics in the published literature, we aimed to collect data based on a planned ST effect and to examine individual differences by recruiting more participants than in previous studies of interest. We were successful in this regard by collecting larger samples than those reported in Schmader and Johns (2003) and Regner et al. (2010); however, we acknowledge that we likely needed even more data considering current replication concerns which were unknown at the time of planning the current study.

5 Controlling for differences in trait and state WMC did not change the effect of ST (the race by condition interaction) on any performance outcomes in the Quantitative Capacity and Intelligence Condition of Experiment 1.

6 For this condition, we also examined ST effects on GRE attempts and anxiety. There was significant main effect of race on the Verbal GRE based on a Bonferroni correction of.0083, *F*(1, 195) = 7.58, *p* =.0065, η_p_^2^ =.0360, *b* =.071, *t*(195) = 3.12, *p* =.0021, CI_95%_ [.026,.116], a main effect of race on the Math GRE, *F*(1, 195) = 9.27, *p* =.0026, η_p_^2^ =.0429, *b* =.086, *t*(195) = 2.61, *p* =.0098 CI_95%_ [.021,.152]. There were no significant effects on anxiety in this condition.

7 The ANOVA models in Experiment 1 were computed with the default setting of Type I SS, whereas the *t* statistic, *b* weight, and CIs were computed from regression models where Type III SS is the default.

8 Controlling for differences in trait and state WMC did not change the effect of ST on any performance outcomes reported in the verbal capacity and intelligence condition of Experiment 1.

9 In this condition, we also examined ST on attempted items on the GRE and on anxiety with and without controlling for trait and state WMC. Not controlling for WMC, and based on a Bonferroni correction on Verbal GRE attempts, there were no significant effects. For the Math GRE attempts, there was a significant interaction, *F*(1, 243) = 3.99, *p* =.047, η_p_^2^ =.016, *b* =.099, *t*(243) = 1.99, *p* =.047, CI_95%_ [.0013,.20]. There were no other significant ST effects on these outcomes in Experiment 1.

10 Each of the ANOVA models in Experiment 1 were computed with the default setting of Type I SS, whereas the *t* statistic, *b* weight, and CIs were computed from regression models (dummy coded with condition 0 = control, 1 = threat, and 0 = White, 1 = Black) where Type III SS is the default.

11 For recent, first-time students at the private university, about 10.12% identified as Hispanic/Latino, 7.7% as Black/African American non-Hispanic, 42.62% as White non-Hispanic, 0.15% as American Indian or Native Alaskan non-Hispanic, 21.44% as Asian non-Hispanic, 0.17% as Native Hawaiian or Pacific Islander non-Hispanic, 4.33% as two or more races, and about 1.47% with race/ethnic identity unknown. About 80.9% scored between 700 and 800 on the Reading and Writing SAT, and 84.19% scored similarly on the Math SAT—indicating a high percentage of students make close to a perfect score (of 1600). For GPA, 88% of students had a GPA of 3.75 or higher.

12 The public state university’s recent freshman class statistics report about 45.77% minority and 53.23% majority students (it is unclear whether minority meant all non-White students). The average combined SAT score was about 1137 and the average weighted GPA reported was 3.61. Because the private university students are recruited based on having very competitive GPAs and standardized test scores, they may have additional practice, preparation, or motivation during standardized tests. By extension, potential differences in these before or during standardized tests, Steele and Aronson (1995) and others (e.g., Steele, 1997) argue that ST should be most harmful for highly motivated students—called the academic “vanguard.” This would suggest that the Black students in the private university sample should be likely to feel and experience the effect of ST on standardized test performance. However, we did not find evidence of this in Experiment 1.

13 Additional data were not collected on age, thus, descriptive information on age was unavailable.

14 For exploratory purposes, a survey battery of noncognitive measures included mindset, personality, and grit.

15 In the quantitative capacity and intelligence condition, we found no significant effects for GRE attempts on either the Verbal or Math GRE. For the anxiety measure, we found a significant interaction in the ANOVA, *F*(1, 77) = 5.96,* p* =.017, η_p_^2^ =.0719. However, based on the 95% CIs, the effects of race, *b* = *− *3.04, *t*(77) = − 2.37, CI_95%_ [− 5.59, −.48], *p* =.020, threat, *b* = *− *3.51, *t*(77) = − 2.73, CI_95%_ [− 6.06, −.95], *p* =.0078, and the interaction, *b* = 4.30, *t*(77) = 2.44, CI_95%_ [.79, 7.80], *p* =.017; effects on anxiety were all significant.

16 Unlike Experiment 1, there were no baseline differences found in Experiment 2 on the OSPAN, so we did not run models to compare results controlling for OSPAN.

17 For the verbal capacity and intelligence condition, there was a significant interaction effect on attempts on the Verbal GRE, *F*(1, 80) = 4.27, *p* =.042, η_p_^2^ =.051, and based on the 95% CIs, the effects of race, *b* =.129, *t*(80) = 2.36, CI_95%_ [.020,.238], *p* =.021, threat, *b* =.144, *t*(80) = 2.53, CI_95%_ [.031,.257], *p* =.013, and the interaction, *b* = *− *.143, *t*(80) = − 2.07, CI_95%_ [−.28, −.0053], *p* =.042, effects were all significant. There were no significant effects on attempts on the Math GRE or on anxiety in this condition.

18 One subject scored perfectly on the Math GRE, was removed, and the analyses were re-run.

19 Selectivity *does not* indicate differences in overall university quality. Instead, the term *selectivity* is used to indicate differences in range of the average standardized test scores and how this maps on to quantitative and/or qualitative differences in student experiences and performance. Selectivity is also germane to differences in weight placed on standardized test scores for admissions purposes.

20 In the quantitative capacity and intelligence condition, for attempts on the Verbal GRE, in spite of a Bonferroni correction of.0083, there was a main effect of race, *F*(1, 277) = 9.33, *p* =.0024, η_p_^2^ =.032. For attempts on the Math GRE, there was a main effect of race, *F*(1, 277) = 4.59, *p* =.033, η_p_^2^ =.016. There were no significant effects on anxiety in this condition.

21 In the verbal capacity and intelligence condition, in spite of the Bonferroni correction of.0083, we found significant effects for race, *F*(1, 332) = 9.69, *p* =.0020, η_p_^2^ =.029, and an interaction, *F*(1, 332) = 12.20, *p* =.00054, η_p_^2^ =.0354 for attempts on the Verbal GRE. Also, with a Bonferroni correction we found a significant interaction effect, *F*(1, 331) = 9.70, *p* =.0020, η_p_^2^ =.028, for attempts on the Math GRE. We did not find any significant effects on anxiety in this condition.

## Code availability

Code to analyze datasets can be made available from the corresponding author on reasonable request.
