# Supplementary material for: Higher trait working memory capacity may benefit standardized test performance under race-related stereotype threat
Source: Mem Cognit. 2025 May 19;53(8):2509–34. doi: 10.3758/s13421-025-01723-y (PMC12695912; doi:10.3758/s13421-025-01723-y)
Supplement: Supplementary file 1 — Supplementary file1 (DOCX 312 KB) [file 13421_2025_1723_MOESM1_ESM.docx]

**Supplemental Information**

**Experiment 1**

**Quantitative Capacity and Intelligence Condition.**

For only Black subjects, a mediation analysis with condition as the independent variable,

OSPAN, the mediator variable, and GRE-M as the outcome variable was conducted. The direct

effect of threat on GRE-M was trending toward significance, *t* (43) =1.8, *b* =.089, *p* =.079. The effect of threat on OSPAN was non-significant, *t* (43) =-.50, *b*=-1.7, *p* = .62. The indirect effect of Threat on GRE-M when OSPAN was added to the model was significant (and increased from

.089 to .099), *t* (42) =2.16, *b*=.0998, *p* = .036, indicating the threat manipulation predicted a .099

increase on GRE-M. In the indirect path, the effect of OSPAN on GRE-M was also significant, *t*

(42) = 2.9, *b =*.0059, *p* = .0059. Moreover, Sobel’s test revealed evidence for statistically

significant mediation of OSPAN in the effect of threat on GRE-M, *Z* = -.49, *p* =.62 (see Figure

1 below).

Using nonparametric bootstrapping methods to resample 1000 simulations, results

indicated non-significant effects for the average causal mediation effect, parameter estimate = -

.0102, *CI* [-.062, .023], *p* = .60, however, the average direct effect of threat on GRE-M was

significant, parameter estimate = .099, *CI* [.014, .19], *p* = .03. Taken together, these findings

reveal a lack of evidence that Black subjects’ WMC (as assessed via the OSPAN) mediated the

effect of threat on GRE-M (see Figure 2 below).

Next, a mediation analysis with condition as the independent variable, OSPAN, the

mediator variable, and GRE-V as the outcome variable was conducted, The direct effect of threat

on GRE-V was not significant, *t* (43) =1.3, *b* = .063, *p* = .21. The effect of threat on

OSPAN was non-significant, *t* (43) = -.50, *b* = -1.7, *p* = .62. The indirect effect of threat on GRE-V when OSPAN was added to the model was not significant (and increased from .063 to .069), *t* (42) =1.5, *b* = .0696, *p* =.16. In the indirect path, the effect of OSPAN on GRE-V was marginally significant, *t* (42) =1.9, *b* =.0039, *p* =.070. Sobel’s test confirmed a lack of strong evidence for statistically significant mediation of OSPAN in the effect of threat on GRE-V, *Z* = -.48, *p* =.63.

Using nonparametric bootstrapping methods to resample 1000 simulations, results

indicated non-significant effects for the average causal mediation effect, parameter estimate =

-.0068, *CI* [-.037, .019], *p* = .60. The average direct effect of threat on GRE-V was also not

significant, parameter estimate=.0696, *CI* [-.035, .165], *p* = .17. Taken together, these findings

reveal a lack of evidence that Black subjects’ WMC (as assessed via the OSPAN) mediated the

effect of threat on GRE-V.


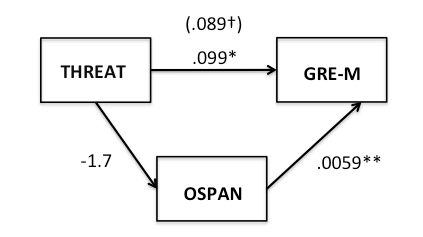


*Figure 1*. OSPAN Mediating Threat Effect on GRE-M.

*Figure 2*. OSPAN Mediation Model for Threat on GRE-M using Bootstrapping.

**Verbal Capacity and Intelligence Condition.**

A mediation analysis for Black subjects with condition as the independent variable,

RSPAN, the mediator variable, and GRE-V as the outcome variable was conducted. The direct

effect of threat on GRE-V was non-significant, *t* (40) =1.08, *b* =.055, *p* = .29. The effect of threat on RSPAN was also non-significant, *t* (40) = -.126, *b* = -.48, *p* = .90. The indirect effect of

Threat on GRE-V when RSPAN was added to the model was non-significant and did not appear

to change much based on including RSPAN in the model, *t* (39) = 1.14, *b* =.057, *p* = .26. In the indirect path, the effect of RSPAN on GRE-V was trending toward significance, *t* (39) =1.8, *b* =.0037, *p* = .084. Moreover, Sobel’s test supported the lack of evidence for statistical mediation of RSPAN in the effect of threat on GRE-V, *Z* = -.12, *p* =.90.

Using nonparametric bootstrapping methods to resample 1000 simulations, results

indicated non-significant effects for the average causal mediation effect, parameter estimate = -

.0018, *CI* [-.038, .032], *p* = .92, as well as the average direct effect of threat on GRE-V,

parameter estimate =.057, *CI* [-.043, .16], *p* = .25. Taken together, these findings reveal a lack of

evidence that Black subjects’ WMC (as assessed via the RSPAN) mediated the effect of threat

on GRE-V.

A mediation analysis for Black subjects with condition as the independent variable,

RSPAN, the mediator variable, and GRE-M as the outcome variable was conducted. The direct

effect of threat on GRE-M was non-significant, *t* (40) =.22, *b* =.013, *p* = .83. The effect of threat on RSPAN was also non-significant, *t* (40) = -.13, *b* = -.48, *p* = .90. In the indirect path the

effect of threat on GRE-M when RSPAN was added to the model was non-significant and did not appear to change much based on including RSPAN in the model, *t* (39) = .22, *b* = .014, *p* =

.82. In the indirect path, the effect of RSPAN on GRE-M was also not significant, *t* (39) = .44, *b*

= .0012, *p* =.66. Moreover, Sobel’s test supported the lack of evidence for statistical mediation of

RSPAN in the effect of threat on GRE-V, *Z* = -.12, *p* =.90.

Using nonparametric bootstrapping methods to resample 1000 simulations, results

indicated non-significant effects for the average causal mediation effect, parameter estimate = -

.00059, *CI* [-.020, .014], *p* = .91, as well as the average direct effect of threat on GRE-M,

parameter estimate=.014, *CI* [-.11, .13], *p* =.87. Taken together, these findings further support a

lack of evidence that Black subjects’ WMC (as assessed via the RSPAN) mediated the effect of

threat on GRE-M. And moreover, resampling results also indicated that the threat manipulation

had a non-significant effect on Black subjects’ performance on the math GRE.

**Experiment 2**

**Quantitative Capacity and Intelligence Condition.**

A mediation analysis for only Black subjects with condition as the independent variable,

OSPAN, the mediator variable, and GRE-M as the outcome variable was conducted. The direct

effect of threat on GRE-M was non-significant, *t* (35) = 1.7, *b* = .045, *p* =.094. The effect of threat on OSPAN was not significant, *t* (35) = 1.2, *b* = 6.97, *p* = .22. The indirect effect of Threat on GRE-M when OSPAN was added to the model was trending toward significant, *t* (34) =1.61, *b* =.044, *p* =.12. In the indirect path, the effect of OSPAN on GRE-M was also non- significant *t* (34) =.26, *b* =.00021, *p* = .80. Sobel’s test further supported the lack of evidence for statistical mediation of OSPAN in the effect of threat on GRE-M, *Z* = .254, *p* =.80.

Bootstrapping methods resampled 1000 simulations to further test for mediation. Results

indicated non-significant effects for the average causal mediation effect, parameter estimate =

.0014, *CI* [-.0148, .0215], *p* = .81, as well as the average direct effect of threat on GRE-M,

parameter estimate=.044, *CI* [-.010, .102], *p* = .11. Taken together, these findings reveal a lack

of evidence that Black subjects’ WMC (as assessed via the OSPAN) mediated the effect of threat

on GRE-M.

A mediation analysis for only Black subjects with condition as the independent variable,

OSPAN, the mediator variable, and GRE-V as the outcome variable was conducted. The direct

effect of threat on GRE-V was non-significant, *t* (35) = -.015, *b* = -.00047, *p* = .98. The effect of

threat on OSPAN was not significant, *t* (35) = 6.97, *b* = 1.24, *p* = .22. The indirect effect of

Threat on GRE-V when OSPAN was added to the model was not significant, *t* (34) = -.06, *b* = -

.0023, *p* = .95. In the indirect path, the effect of OSPAN on GRE-V was also not significant *t*

(34) = .27, *b* =.00026, *p* = .27. Sobel’s test further supported the lack of evidence for statistical

mediation of OSPAN in the effect of threat on GRE-V, *Z* = .26, *p* = .79.

Bootstrapping methods resampled 1000 simulations to further test for mediation. Results

indicated non-significant effects for the average causal mediation effect, parameter estimate =

-.0068, *CI* [-.037, .023], *p* = .63, as well as the average direct effect of threat on GRE-V,

parameter estimate =.0696, *CI* [-.030, .163], *p* =.15. Taken together, these findings reveal a lack

of evidence that Black subjects’ WMC (as assessed via the OSPAN) mediated the effect of threat

on GRE-V.

**Verbal Capacity and Intelligence Condition.**

A mediation analysis for only Black subjects with condition as the independent variable, RSPAN, the mediator variable, and GRE-V as the outcome variable was conducted. The direct effect of threat on GRE-V was non-significant, *t* (29) = .93, *b* =.036, *p* = .36. The effect of threat on RSPAN was trending toward significant, *t* (29) = -1.93, *b* = -11.45, *p* = .063. The indirect effect of Threat on GRE-V when RSPAN was added to the model was non-significant, *t* (28) = .36, *b* = .015, *p* = .72. In spite of the non-significant effect, a decrease in the coefficient for threat (from *b*=.036, to *b*=.015) was observed, indicating that the effect of stereotype threat on verbal GRE was at least partially reduced as a result of including RSPAN in the model. In the indirect path, the effect of RSPAN on GRE-V was also non- significant *t* (28) = -1.58, *b* = -.0019, *p* = .13. Sobel’s test further supported the lack of strong evidence for statistical mediation of RSPAN in the effect of threat on GRE-V, *Z* = 1.22, *p* =.22.

Using nonparametric bootstrapping methods to resample 1000 simulations, results indicated non-significant effects for the average causal mediation effect, parameter estimate =

.021, CI_95%_ = [-.0098, .067], *p* =.21, as well as the average direct effect of threat on GRE-V, parameter estimate=.015, CI_95%_ = [-.055, .095], *p* =.74. Taken together, these findings reveal a lack of evidence that Black subjects’ WMC (as assessed via the RSPAN) mediated the effect of threat on GRE-V.

Next, a mediation analysis for only Black subjects with condition as the independent variable, RSPAN, the mediator variable, and GRE-M as the outcome variable was conducted. The direct effect of threat on GRE-M was non-significant, *t* (28) = 1.34, *b* =.052, *p* = .19. The effect of threat on RSPAN was trending toward significant, *t* (28) = -1.62, *b* = -9.9, *p* =.12. The indirect effect of Threat on GRE-M when RSPAN was added to the model was marginally significant, *t* (27) =1.97, *b* =.075, *p* =.06 indicating an increase in the coefficient for threat (from *b*=.052, to *b*=.075) was observed. In the indirect path, the effect of RSPAN on GRE-M was significant *t* (27) = 2.08, *b* =.0024, *p* = .047. Moreover, Sobel’s test further supported the lack of strong evidence for statistical mediation of RSPAN in the effect of threat on GRE-V, *Z* = -1.28, *p* =.20 (see Figure 3).

Using nonparametric bootstrapping methods to resample 1000 simulations, results indicated a marginally significant effect for the average causal mediation effect, parameter estimate = -.023, *CI* [-.064, .0031], *p* =.10. The average direct effect of threat on GRE-M was significant, parameter estimate =.075, *CI* [-.0051, .145], *p* =.04, suggesting that the effect of threat on GRE-M here predicted a small increase (not a performance decrement). Taken together, these findings reveal a lack of clear evidence that Black subjects’ WMC (as assessed via the RSPAN) mediated the effect of threat on GRE-M. Moreover, if anything the effect of threat appeared to go in the opposite direction of the prediction (see Figure 4 below).

*Figure 3*. RSPAN Mediating Threat Effect on GRE-M.

*Figure 4*. RSPAN Mediation Model for Threat on GRE-M using Bootstrapping.

**Combined Analysis**

**Quantitative Capacity and Intelligence Condition.**

For Black subjects in the combined samples, a mediation analysis with condition as the independent variable, OSPAN, the mediator variable, and GRE-M as the outcome variable was conducted. The direct effect of threat on GRE-M was not significant, *t* (80) = 1.63, *b* = .066, *p* = .11. The effect of threat on OSPAN was non-significant, *t* (80) = .62, *b* = 2.07, *p* = .53. The indirect effect of Threat on GRE-M when OSPAN was added to the model was not significant, *t* (79) = 1.49, *b* = .057, *p* =.14. In the indirect path, the effect of OSPAN on GRE-M was significant, *t* (79) = 3.41, *b=*.0044, *p* = .001. Moreover, Sobel’s test revealed a lack of evidence for statistically significant mediation of OSPAN in the effect of threat on GRE-M, *Z* =.61, *p* = .54.

Using nonparametric bootstrapping methods to resample 1000 simulations, results

indicated non-significant effects for the average causal mediation effect, parameter estimate=-

.0090, *CI* [-.020, .04], *p* =.50, the average direct effect of threat on GRE-M was

also not significant, parameter estimate=.057, *CI* [-.021, .13], *p* =.13. Taken together, these findings reveal a lack of evidence that Black subjects’ WMC (as assessed via the OSPAN) mediated the effect of threat on GRE-M.

Next, a mediation analysis with condition as the independent variable, OSPAN, the

mediator variable, and GRE-V as the outcome variable was conducted. The direct effect of threat on GRE-V was not significant, *t* (80) = .67, *b* = .030, *p* = .50. The effect of threat on

OSPAN was non-significant, *t* (80) = .62, *b* = 2.07, *p* = .53. The indirect effect of threat on GRE-V when OSPAN was added to the model was not significant, *t* (79) =.50, *b* =.021, *p* = .62. In the indirect path, the effect of OSPAN on GRE-V was significant, *t* (79) = 2.89, *b* = .0041, *p* = .0050. Sobel’s test revealed no statistically significant mediation of OSPAN in the effect of threat on GRE-V, *Z* = .61, *p* = .54.

Using nonparametric bootstrapping methods to resample 1000 simulations, results

indicated non-significant effects for the average causal mediation effect, parameter estimate =

.0086, *CI* [-.019, .04], *p* = .49. The average direct effect of threat on GRE-V was also not

significant, parameter estimate=.021, *CI* [-.059, .11], *p* =.61. Taken together, these findings

reveal a lack of evidence that Black subjects’ WMC (as assessed via the OSPAN) mediated the

effect of threat on GRE-V.

**Bayesian Regression Mediation**

We also ran Bayesian Regression analyses to examine the mediation effects.

We used the Bayes Factor package in R (Morey & Rouder, 2015) to compute Bayes Factors for direct effects only models and the indirect effects models. Each of these was run compared to a null or intercept only model. To test for mediation, we then computed the Bayes Factors for the indirect effects only model and compared those to the direct effect model. These results are discussed in more detail below.

The full model results for the Quantitative Capacity and Intelligence condition are included in Table SI 1 below. In the mediation model predicting math GRE the Bayes Factor for the direct effect of OSPAN revealed very strong evidence relative to the null (*BF* = 37.48). The direct effect of threat condition contained a Bayes Factor less than 1 (*BF* = .722) indicating support for the null. In the model comparison for the effect of OSPAN mediating the effect of threat condition on the math GRE the Bayes Factor (*BF* = 35.88) found very strong evidence in favor of the indirect effect compared to the direct effect only.

In the mediation model predicting verbal GRE the Bayes Factor (*BF* = 8.92) for the direct effect of OSPAN revealed strong evidence against the null. The Bayes Factor for the direct effect of threat condition contained a Bayes Factor less than 1 (*BF* = .28) indicating support for the null. In the model comparison for the effect OSPAN mediating the effect of threat condition on the verbal GRE, the Bayes Factor (*BF* = 9.93) found very strong evidence in favor of the indirect effect compared to the direct effect only.

**SI Bayesian Mediation Summary Table 1**

**Verbal Capacity and Intelligence Condition.**

Next, a mediation analysis with condition as the independent variable, RSPAN, the

mediator variable, and GRE-V as the outcome variable was conducted. The direct effect of threat on GRE-V was not significant, *t* (69) = -.102, *b* = -.0051, *p* = .92. The effect of threat on RSPAN was non-significant, *t* (69) = -1.21, *b*= -3.76, *p* = .23. The indirect effect of threat on GRE-V when RSPAN was added to the model was not significant, *t* (68) =.14, *b* =.0072, *p* =.89. In the indirect path, the effect of RSPAN on GRE-V was marginally significant, *t* (68) = 1.70, *b* = .0033, *p* = .093. Sobel’s test confirmed a lack of evidence for statistically significant mediation of RSPAN in the effect of threat on GRE-V, *Z* = -.98, *p* = .32.

Using nonparametric bootstrapping methods to resample 1000 simulations, results

indicated non-significant effects for the average causal mediation effect, parameter estimate =

-.012, *CI* [-.042, .01], *p* =.27. The average direct effect of threat on GRE-V was also not

significant, parameter estimate=.0072, *CI* [-.092, .10], *p* =.10. Taken together, these findings

reveal a lack of evidence that Black subjects’ WMC (as assessed via the RSPAN) mediated the

effect of threat on GRE-V.

Next, a mediation analysis was run with GRE-M as a dependent measure. The direct effect of threat on GRE-M was not significant, *t* (69) = -.029, *b* = -.0014, *p* = .98. The effect of threat on RSPAN was non-significant, *t* (69) = -1.21, *b* = -3.76, *p* = .23. The indirect effect of threat on GRE-M when RSPAN was added to the model was not significant, *t* (68) =.15, *b* =

.0071, *p* =.88. In the indirect path, the effect of RSPAN on GRE-M was not significant, *t* (68) = 1.24, *b* = .0023, *p* =.22. Sobel’s test confirmed a lack of evidence for statistically significant mediation of RSPAN in the effect of threat on GRE-M, *Z* = -.87, *p* =.39.

Using nonparametric bootstrapping methods to resample 1000 simulations, results

indicated non-significant effects for the average causal mediation effect, parameter estimate =

-.0085, *CI* [-.035, .00], *p* =.31. The average direct effect of threat on GRE-M was also not

significant, parameter estimate=.0071, *CI* [-.085, .10], *p* = .82. Taken together, these findings

reveal a lack of evidence that Black subjects’ WMC (as assessed via the RSPAN) mediated the

effect of threat on GRE-M.

**Bayesian Regression Mediation**

We also ran Bayesian Regression to examine the mediation effects for the Verbal Capacity and Intelligence condition and the full model results are included in Table SI 2 below. In the mediation model predicting verbal GRE neither the direct effects of RSPAN nor threat condition contained Bayes Factors greater than 1 (*BF* = .85 and *BF* = .245, respectively) indicating support for the null in both cases. In the model comparison for the effect RSPAN mediating the effect of threat condition on the verbal GRE, the Bayes Factor (*BF* = 1.16) found anecdotal evidence in favor of the indirect effect compared to the direct effect only.

In the mediation model predicting math GRE neither the direct effects of RSPAN nor threat contained Bayes Factors greater than 1(*BF* = .472 and *BF* = .245, respectively) indicating support for the null in both cases. In the model comparison for the effect RSPAN mediating the effect of threat condition on the math GRE, the Bayes Factor was less than 1 (*BF* = .669) indicating very weak evidence in favor of the indirect effect compared to the direct effect only.

**SI Bayesian Mediation Summary Table 2**
